# Catalytic Asymmetric α-Functionalization of α-Branched Aldehydes

**DOI:** 10.3390/molecules28062694

**Published:** 2023-03-16

**Authors:** Silvia Vera, Aitor Landa, Antonia Mielgo, Iñaki Ganboa, Mikel Oiarbide, Vadim Soloshonok

**Affiliations:** 1 Department of Organic Chemistry I, University of the Basque Country UPV/EHU, Manuel Lardizabal 3, 20018 Donostia-San Sebastián, Spain; 2 IKERBASQUE, Basque Foundation for Science, Plaza Euskadi 5, 48009 Bilbao, Spain

**Keywords:** aldehydes, asymmetric catalysis, quaternary carbon, organocatalysis, dual catalysis, reaction umpolung

## Abstract

Aldehydes constitute a main class of organic compounds widely applied in synthesis. As such, catalyst-controlled enantioselective α-functionalization of aldehydes has attracted great interest over the years. In this context, α-branched aldehydes are especially challenging substrates because of reactivity and selectivity issues. Firstly, the transient trisubstituted enamines and enolates resulting upon treatment with an aminocatalyst or a base, respectively, would exhibit attenuated reactivity; secondly, mixtures of *E*- and *Z*-configured enamines/enolates may be formed; and third, effective face-discrimination on such trisubstituted sp^2^ carbon intermediates by the incoming electrophilic reagent is not trivial. Despite these issues, in the last 15 years, several catalytic approaches for the α-functionalization of prostereogenic α-branched aldehydes that proceed in useful yields and diastereo- and enantioselectivity have been uncovered. Developments include both organocatalytic and metal-catalyzed approaches as well as dual catalysis strategies for forging new carbon–carbon and carbon–heteroatom (C-O, N, S, F, Cl, Br, …) bond formation at Cα of the starting aldehyde. In this review, some key early contributions to the field are presented, but focus is on the most recent methods, mainly covering the literature from year 2014 onward.

## 1. Introduction

Compounds possessing a tetrasubstituted carbon stereocenter are widely found within natural products, as well as bioactive substances. As a consequence, the construction of synthetically versatile building blocks bearing a tetrasubstituted carbon stereocenter in a fully stereocontrolled manner has attracted considerable attention [1,2,3,4,5,6,7,8,9,10]. In this context, aldehydes not only are widely accessible starting materials, but also represent powerful intermediates in organic synthesis. The development of catalyst-controlled sustainable methods for the preparation of aldehydes with a quaternary stereogenic α-carbon is therefore highly desirable. With the irruption of aminocatalytic methods for the direct, asymmetric α-functionalization of both aldehydes and ketones, huge advances were made during the first decade of this century that uncovered methods for the direct formation of new carbon–carbon and carbon–heteroatom (heteroatom= halogen, nitrogen, oxygen, and sulfur) bonds at the aldehyde α-carbon enantioselectively via transient enamine intermediates [11,12,13,14,15]. While the enamine activation mode has proved quite successful for the enantioselective α-functionalization of linear aldehydes [16,17,18,19,20,21,22,23], its extension to α-branched aldehydes progresses with paucity. In response to this situation, complementary activation modes have entered the field, including methods based on dual activation strategies [24,25,26,27,28,29,30,31,32,33,34]. In 2014, Moreau and coworkers published a major review entitled *asymmetric organocatalytic functionalization of α,α-disubstituted aldehydes through enamine activation* [35]. Since then, new methods based on enamine-mediated reactions and complementary ones using alternative activation modes, including methods starting from nonaldehyde precursors such as alkene hydroformylation [36,37,38,39,40], have appeared which have helped to provide enantioenriched α-tetrasubstituted aldehydes as useful synthons for synthesis. In this review, we present the state of the art in α-functionalization of α-branched aldehydes, highlighting the requirements and scope of each method, with a focus on the substrate activation mechanism(s).

**Scheme 1:**
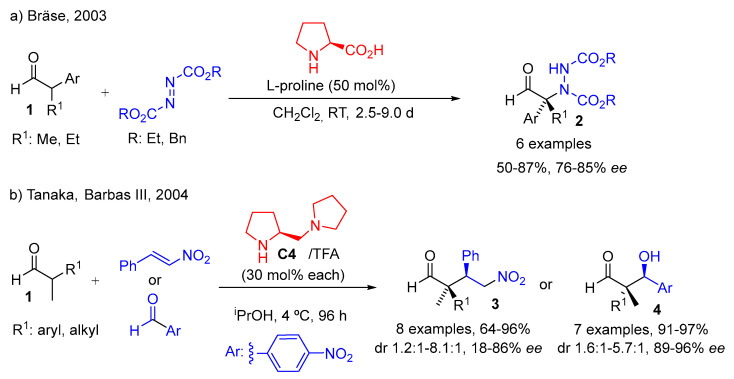
Pioneering reports on catalytic asymmetric α-functionalization of α-branched aldehydes.

Asymmetric α-functionalization of α-branched aldehydes was first reported in 2003 by the group of Bräse, who described the proline-catalyzed electrophilic amination of α-branched propionaldehydes and butyraldehydes **1** with dialkyl azodicarboxylates (Scheme 1) [41,42,43]. In the initial development, 50 mol% of catalyst was required along with long reaction times, and the α-aryl aldehydes gave rise to α-hydrazino aldehydes **2** in moderate to good yields and with variable levels of enantiocontrol. Furthermore, the reactions with the α,α-dialkyl substituted aldehydes gave impractical results. Soon thereafter, Tanaka, Barbas III and coworkers reported the first enantioselective C-C bond forming additional reactions of α-branched prostereogenic aldehydes using a 2-pyrrolidinyl-pyrrolidine catalyst **C4** with trifluoroacetic acid cocatalyst [44,45].

While only modest catalyst efficiency as well as enantiocontrol were achieved in these initial developments, the research data revealed the difficulties inherent to enamine formation from α-branched aldehydes and stereocontrol issues due to the fact that both *E* and *Z*-configured enamines may be formed. Nevertheless, the viability of the approach was clearly demonstrated. In subsequent studies by these and other research groups, new aminocatalysts, particularly primary amine catalysts, were introduced, allowing for noticeable improvement of the reaction outcomes in these and related α-carbo and α-heterofunctionalization (α-oxylation, α-fluorination, and α-chlorination) processes. Progress in this field that covered the literature until the year 2013 has been already reviewed [35]. In the current review, selected key early contributions are described in some detail, but focus is made on the most recent advances from that date onward. Aminocatalysts developed in that initial period of years often continue to be employed in some newer methods too, but the assistance of a different co-catalyst or additive, or just the use of modified conditions, has allowed for the accomplishment of new achievements in the area which were inaccessible previously by simple enamine activation approaches. In recent years, alternative activation modes have also been uncovered that could be applied to the enantioselective α-functionalization of α-branched aldehydes. Here, this increasingly broader collection of approaches is organized based on the mode of substrate activation (e.g., catalysis type), starting with single activation protocols, then moving to bifunctional and dual activation methods.

## 2. Methods Based on Enamine Activation

**Figure 1:**
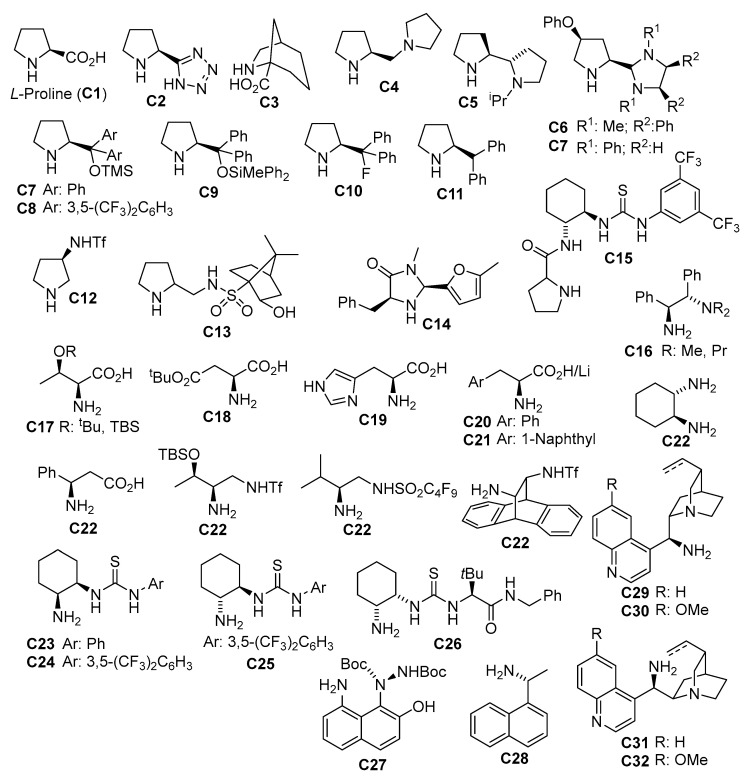
Representative primary and secondary amine catalysts used for enamine-mediated synthesis of α-quaternary aldehydes until the year 2014.

Figure 1 shows a compilation of primary and secondary amine catalysts used for enamine-mediated enantioselective synthesis of α-quaternary aldehydes until ca. year 2014.

(i) α-Heterofunctionalization (oxylation, amination, fluorination, chlorination, and thiolation)

**Scheme 2:**
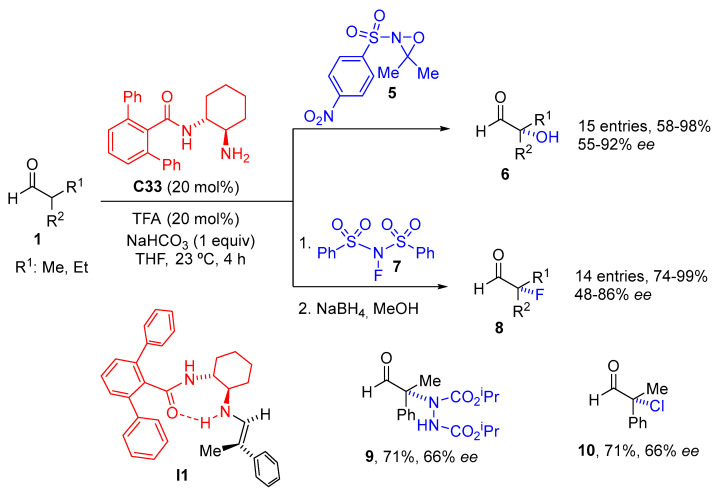
Enamine-mediated α-amino, α-oxy, α-thio, and α-halo-functionalization of aldehydes.

Aminocatalysis has demonstrated to be well-suited for the asymmetric α-heterofunctionalization of α-branched aldehydes with an array of oxidation, amination, halogenation, and thiolation reagents. In particular, the area of organocatalytic asymmetric fluorination has attracted exceptionally high interest due to the growing importance of fluorinated compounds in the design of modern pharmaceuticals [46,47,48]. In 2015, Jacobsen reported the catalytic enantioselective α-hydroxylation and α-fluorination of prostereogenic α-branched aldehydes based on a primary amine-amide bifunctional catalyst **C33** (Scheme 2) [49]. Secondary amine catalysts are inefficient for most reactions involving α-branched aldehydes. For that reason, primary amines are the catalyst of choice, but issues arise concerning the control of the *E/Z* geometry of the formed enamine intermediate and tautomer isomerization. Jacobsen designed catalyst **C33** to solve these problems and found that it was active in oxidations using the *N*-sulfonyl oxaziridine reagent **5** (Yoon oxaziridine) and the commercially available NFSI reagent **7**. During reaction optimization, primary amine-thiourea and urea catalysts were also screened, but they led to slightly lower enantioselectivity than the simple amine-amide **C33**, proving that the dual H-bond donor was unnecessary for these reactions. α-Aryl propionaldehydes are oxidized, affording the α-hydroxy derivatives **6** in good yields and enantioselectivities from good to very good. The reaction also tolerated both propanal and butanal with a benzylic substituent at Cα, although these challenging substrates led to diminished enantioselectivities. The α-fluorination reaction (products **8**) using the same catalyst usually proceeded with slower enantioselectivity than the α-hydroxilations (products **6**), but product **8** enantiopurity could be upgraded conveniently by simple recrystallization. Both reactions could be scaled up to gram quantities with no erosion in both yield and selectivity. Moreover, the present catalytic systems were also demonstrated as successful for the corresponding α-chlorination (products **10**) and α-hydrazination (products **9**) reactions.

**Scheme 3:**
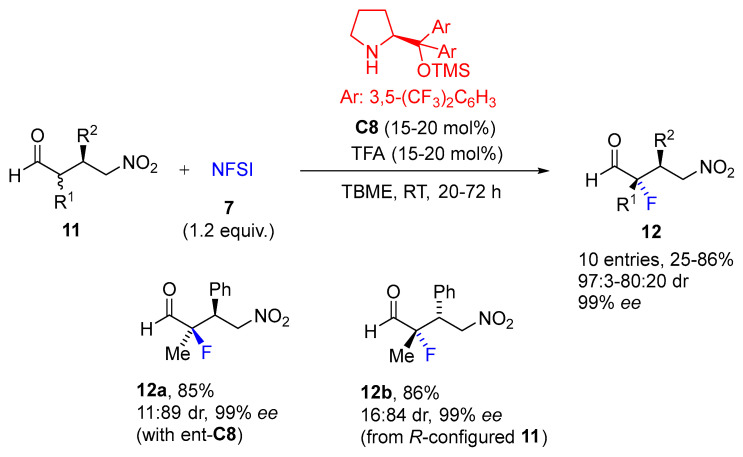
α-Fluorination of chiral γ-nitroaldehydes.

Lombardo-Quintavalla and coworkers studied the catalytic enantioselective α-fluorination reaction of α-branched γ-nitroaldehydes as specific substrates, which are accessed as essentially enantiopure compounds from the amine-catalyzed addition of the corresponding aldehyde to nitroolefins (Scheme 3) [50]. This α-fluorination was not trivial because previous α-fluorinations of aldehydes involving the α,α-dialkyl substituted congeners led to diminished enantioselectivities. In addition, since the substrates bear a γ-stereocenter, whether this center may interfere in the reaction and finding possible matching and mismatching substrate-catalyst pairs presented additional issues. After screening a large collection of chiral pyrrolidines and oxazolidinones as catalysts, as well as additives and solvents, the fluorination reaction with NFSI best proceeded using the Hayashi-Jørgensen catalyst **C8** in 15–20 mol% loading with the same amount of TFA in TBME as the solvent (Scheme 3). Adducts **12** were obtained with essentially perfect enantioselectivity and very good diastereomeric ratios in most cases, including aldehydes with a branched alkyl substituent larger than methyl. Control experiments using the enantiomeric pyrrolidine catalyst ent-**C8**, starting from the *S*-configured nitro compound, which led respectively to adducts **12a** and **12b**, demonstrated that the stereochemistry of the fluorination is controlled by the catalyst, and the effect of the pre-established γ-stereocenter is marginal. Mechanistic studies also revealed that the fluorination step is irreversible and that the *E/Z* equilibration is quite slow under the catalytic conditions. Furthermore, although the *Z*-enamine proved to be more reactive than *E*-enamine, due to the very low concentration of the former in solution, authors concluded that the reaction proceeds via *E*-enamine mainly.

**Scheme 4:**
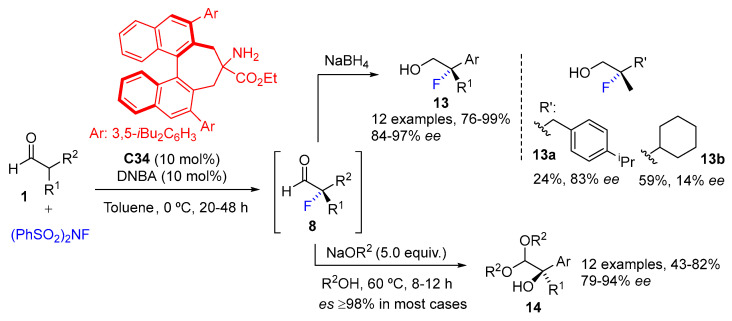
An unnatural α-minoester-catalyzed aldehydes α-fluorination.

Shibatomi described a highly enantioselective fluorination of α-branched aldehydes with *N*-fluoro bis(phenylsulfonyl)imide using the newly developed chiral primary amine **C34** in combination with 3,5-dinitrobenzoic acid as the cocatalyst (Scheme 4) [51]. The α-fluorinated aldehydes were difficult to purify, and instead they were reduced with NaBH_4_ in situ to give rise to alcohols **13**. The α-methyl-alkyl aldehydes led to diminished yields (product **13a**) or selectivity (product **13b**). Alternatively, aldehydes **1** could also be transformed into α-hydroxyacetals **14** in high enantiomeric purity through a stereospecific C-F bond cleavage and ketalization.

**Scheme 5:**
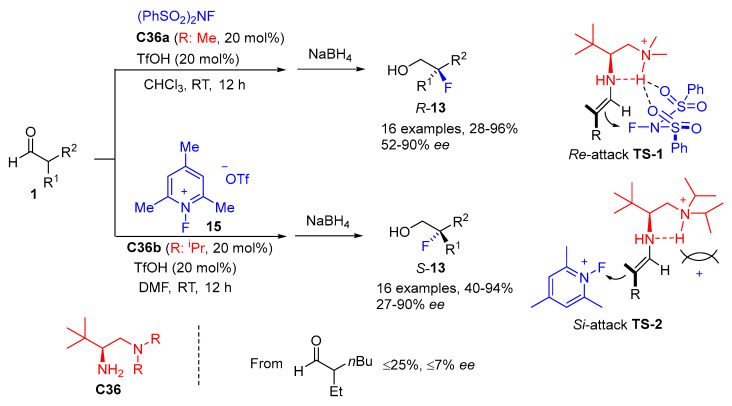
Reagent-dependent enantioswitch of the aldehyde α-fluorination.

Mi, Luo and coworkers developed an enamine-mediated asymmetric fluorination of α--branched aldehydes featuring a reagent-controlled enantioselectivity switch (Scheme 5) [52]. Under optimized conditions, fluorination of α-aryl-propionaldehydes using *N*-fluoro bis(sulfonyl)imide **2** in chloroform in the presence of primary amine **C36a** and TfOH as additives afforded, upon reduction with NaBH_4_, *R*-configured fluoroalcohols *R*-**13** as the major isomer in generally good yields and enantioselectivities. In contrast, fluorination with pyridinium fluoride **15** in DMF in the presence of primary amine **C36b** afforded *S*-configured fluoroalcohols *S*-**13** as the major isomer. As usual, fluorination reactions involving alkyl,alkyl-substituted aldehydes (e.g., 2-ethylhexanal) were less efficient in terms of both chemical yields and enantiocontrol. Authors proposed the rational for the observed enantioswitch based on an attractive H-bonding interaction between the catalyst-protonated amine moiety and the fluorinating reagent S=O groups (*Re*-attack leading to *R*-product, **TS-1**), while repulsive electrostatic interaction with fluorinating reagent **15** would prevent *Re*-face approaching (**TS-2**).

**Scheme 6:**
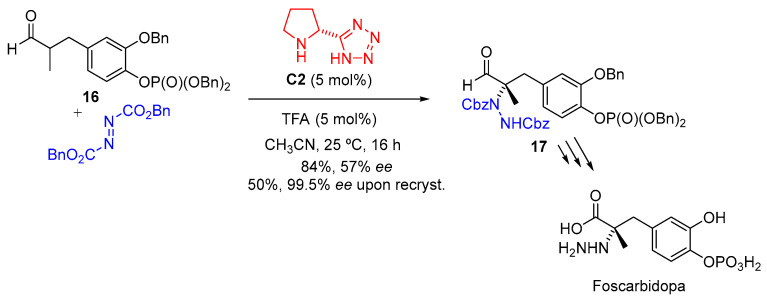
α-Hydrazination of aldehyde applied to synthesis.

In a recent variation of the aminocatalytic α-hydrazination of α-branched aldehydes pioneered by Brässe and Barbas, independently, an industrial group at AbbVie Inc. (North Chicago, IL, USA) has reported the α-hydrazination of aldehyde **16** as a key step in the total synthesis of foscarbidopa, a phosphate prodrug of carbidopa for the treatment of Parkinson’s disease (Scheme 6) [53]. Under optimized conditions using tetrazole-proline catalyst **C2** and TFA as additives in acetonitrile, the hydrazine-aldehyde **17** was obtained in 84% yield and 57% *ee*. Ulterior crystallization from water allowed to obtain essentially enantiopure material in 50% yield that was converted into foscarbidopa after two additional high-yielding steps.

**Scheme 7:**
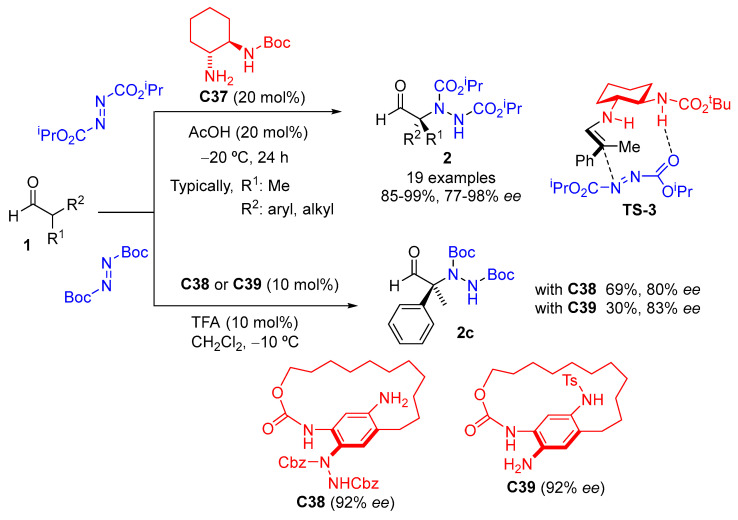
Primary amine-catalyzed α-hydrazination of aldehydes.

Very recently, Gómez-Bengoa, Chinchilla and co-workers reported a solvent-free catalytic α-hydrazination of branched aldehydes with azodicarboxylates in the absence of solvents that proceeds in usually high yields and enantioselectivities using 20 mol% of the primary amine catalyst **C37** and acetic acid as the cocatalyst (Scheme 7) [54]. The bifunctional activation mode imparted by the new catalyst (**TS-3**) was supported on computational DFT studies. As compared with other methods, this protocol is general for α-aryl and α-alkyl propionaldehydes, and even other α-branched aldehydes such as 2-phenylbutanal and tetrahydronaphthalene 2-carbaldehyde provided good results. In addition, the process could be run at 3 mmol scale without loss of yield and enantioselectivity. On the other hand, Xue, Yang and coworkers have applied planar-chiral aniline macrocycles **C38** and **C39** to the α-hydrazidation of 2-phenylpropanal with *N*-Boc hydrazines to afford compound **2c** in moderate yields and high enantioselectivity. It should be noted that both catalysts **C38** and **C39** were not enantiopure [55].

**Scheme 8:**
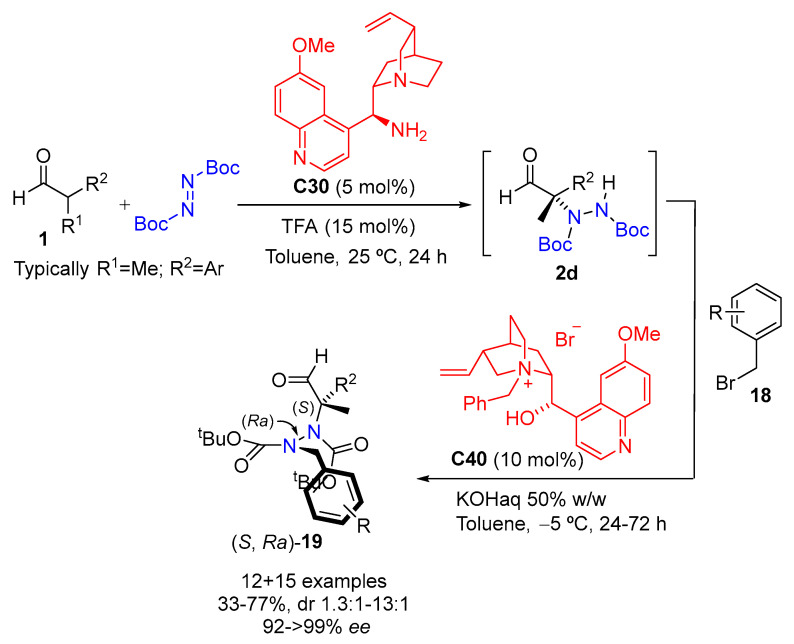
Sequential α-hydrazination/*N*-alkylation towards atropisomeric hydrazines.

As part of their new enantioselective approach to atropisomeric hydrazides, Bencivenni and coworkers reported the asymmetric α-hydrazination of branched aldehydes catalyzed by primary amine **C30**. The one-pot *N*-alkylation of the resulting NH hydrazides **2d** under PTC conditions using catalyst **C40** led to hydrazides **19** featuring a rotationally restricted *N*-*N* bond that may present axial chirality (Scheme 8) [56]. Products **19** with (*S*, *Ra*) configuration are obtained in generally good diastereomeric ratios and excellent enantioselectivity. Interestingly, permutations of organocatalysts **C30** and **C40** with their enantiomers allowed access to the four possible stereoisomeric products.

**Scheme 9:**
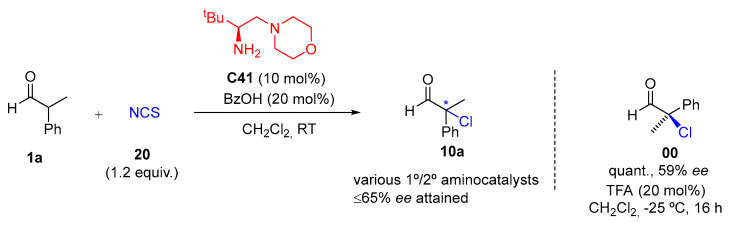
Aldehydes α-chlorination and the origin of low stereoselectivity.

In an interesting study, the Jørgensen group reexamined the amine-catalyzed α-chlorination of 2-phenylpropanal with the aim to clarify the origin of the moderate enantioselectivities usually accompanying this α-halogenation process regardless of the source of “Cl+” used (Scheme 9) [57]. Several primary, secondary, and even tertiary chiral amines were screened with benzoic acid as an additive. The majority of them afforded the desired α-chloro tetrasubstituted aldehyde with yields from low to very high, but poor enantioselectivity (37% *ee* at best). The reaction catalyzed by the morpholine-derived primary amine **C41** was the most promising. After reoptimization of the conditions for this catalyst, product **10a** could be obtained with 85% yield and 63% *ee* in dichloromethane after 16 h at −20 °C (92% yield and 65% *ee* in toluene at −20 °C after a prolonged time (72 h)). No significant variation was observed when chlorinating reagents other than *N*-chlorosuccinimide, selected as reference, were employed. Control experiments suggested that neither racemization of the chlorinated product formed nor resolution of it by re-condensation of the catalyst exerted a significant impact on the enantioselectivity. While some alternative explanations were also considered and ruled out, the experimental and theoretical (computational) results support the hypothesis that the lack of stereoselectivity originates from poor control in the formation of the reactive enamine intermediates. In addition, a clustering of reaction barriers for the various possible reaction pathways found computationally may explain the difficulties in obtaining high enantioselectivity on the α-chlorination reaction under study.

**Scheme 10:**
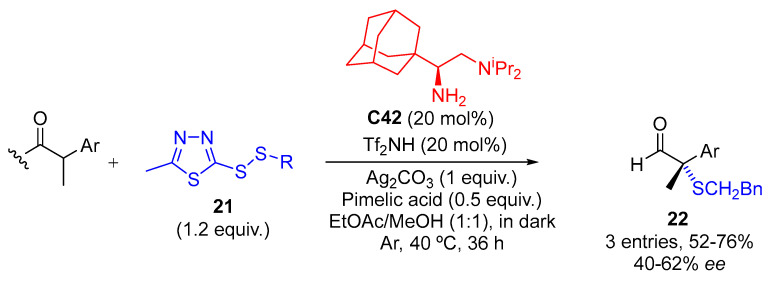
α-Alkylthiolation of α-branched aldehydes.

Mi, Luo and coworkers have recently developed a new alkylthiolation reagent **21** which was successfully employed for the enamine-mediated enantioselective α-alkylthiolation of α-branched aldehydes among other enolizable substrates (Scheme 10) [58]. The use of one equivalent of silver carbonate was critical for a clean reaction, as well as the presence of an acid (e.g., pimelic acid) additive for an efficient reaction. Three α-aryl propionaldehydes were benzylthiolated under these conditions, giving rise to adducts **22** in yields from moderate to good and moderate enantioselectivity using the adamantyl-derived amine **C42**.

(ii) α-alkylation reactions

**Scheme 11:**
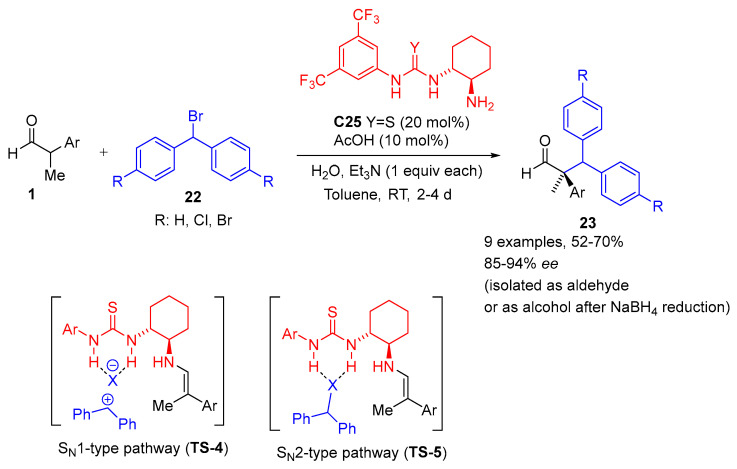
Diarylmethylation of α-branched aldehydes catalyzed by thiourea-primary amines.

The majority of aminocatalysts—from proline and other simple α-amino acids to more elaborated aminocatalysts—employed in the enamine-mediated enantioselective α-functionalization of α-branched aldehydes bear a pending H-bond donor side-arm, which plays a key role by concomitantly activating the electrophile through H-bonding and rigidifying the transition state of the reaction. The bifunctional nature of these catalysts has been demonstrated to be the key for both catalytic efficiency and effective asymmetric induction. In this context, the thiourea/primary amine catalysts introduced by Jacobsen for the α-alkylation of aldehydes with benzhydryl bromides and chlorides constituted a different category of bifunctional catalysis. Here, the thiourea moiety induces the reaction by halide anion abstraction to generate a carbocationic intermediate that is intercepted by the in situ-formed enamine (Scheme 11) [59]. The reaction between α-aryl acetaldehydes **1** and benzhydryl halides **22** required 20 mol% of thiourea catalyst **C25** along with 1 equivalent of triethylamine as an acid scavenger (HBr or HCl evolves). As it happens in other enamine mediated processes, the addition of controlled amounts of water and a catalytic amount of AcOH as weak Brønsted acid accelerated the reaction. The parent urea catalyst was found to be less efficient for this transformation, while more elaborate primary aminothiourea catalysts bearing additional stereogenic units afforded no apparent advantage.

Mechanistic investigations were insightful and served to discern between the two conceivable pathways, namely the S_N_1- and S_N_2-type mechanisms as represented by concurrent activation modes **TS-4** and **TS-5**, respectively (Scheme 11). Secondary kinetic isotope effect (k_H_/k_D_ = 1.12) at the benzhydryl position as well as good Hammett correlation between the halide electronic properties and reactivity were found, which strongly support the S_N_1-type mechanism, involving a catalyst-associated carbocation intermediate. Additional evidences in favor of a catalyst-induced S_N_1-type mechanism were found in competitive experiments using benzyl bromide as a potential electrophile in the alkylation reaction. In a comparison experiment using α-phenylpropanal, benzhydryl chlorides and bromides led to a very similar enantioselectivity.

**Scheme 12:**
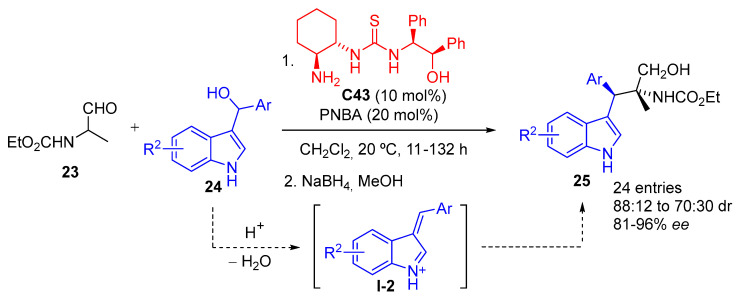
Enantioselective reactions of α-amino aldehydes with indolyl-aryl-methanols.

Active biaryl methanols are green alkylating reagents susceptible to reactions with nucleophilic carbon species with the evolution of a water molecule. Among several other carbon nucleophiles, enamines as transient species derivable from aldehydes have been found capable to react with biaryl methanols [60]. In this context, in 2014, Guo reported a variant involving α-amino aldehydes as nucleophilic components that react with 3-indolylmethanols in the presence of primary amine-thiourea catalysts **C43** (Scheme 12) [61]. During the exploration of the best performing amine catalyst, secondary amines such as proline were expectedly inefficient in promoting the reaction. Various amine-thiourea species were able to catalyze the reaction with variable levels of both enantio- and diastereoselectivity. Thiourea **C43**, derivable from (1*R*,2*R*)-2amino-1,2-diphenylethanol, promoted the reaction with an acceptable diastereoselection (typically around 4:1) and high enantioselectivity for the major diastereomer **25**. Optimization of the reaction conditions allowed for the identification of *p*-nitrobenzoic acid as the optimum acid cocatalyst (20 mol%) required to promote the formation of the iminium species **I-2**, and the ethyl carbamate as the best protecting group of the amino group on starting α-amino propanaldehyde. The reaction was particularly sensitive to the size of the aldehyde α-substituent, with groups larger than methyl providing diminished yields. The resulting indol-derived adducts, which were isolated as the corresponding primary alcohol **25** upon in situ treatment with sodium borohydride, may be used in several synthetic applications. Extension of this approach to less active bisarylmethanols showed to be problematic.

**Scheme 13:**
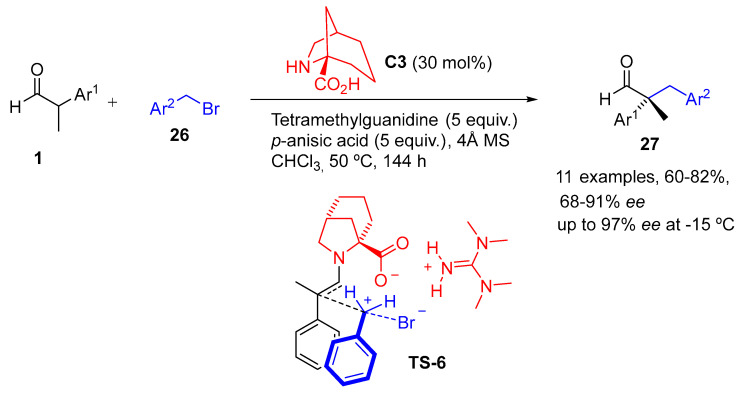
α-Amino acid-catalyzed C-benzylation of branched aldehydes.

List developed the first aminocatalyzed α-alkylation of α-branched aldehydes with benzyl bromides [62]. In this transformation, besides the usual problems of amine-promoted aldehydes self-aldolization and racemization, the tendency of Lewis basic amine catalysts to undergo irreversible *N*-alkylation with S_N_2-type alkylating agents may prevail. Authors rationalized that these potential shortcomings may be minimized if branched aldehydes in combination with an organic mixed acid/base “buffer” system is used instead of a base alone. Indeed, initial experiments with hydratropaldehyde and benzyl bromide as the model reaction in the presence of a mixture of *p*-anisic acid and diisopropylethylamine afforded the desired α-quaternary aldehyde product, although in yet unsatisfactory yield and selectivity. Further screening of catalysts and conditions showed that by using azabicyclic proline analogs such as **C3**, which were known to provide better preorganization of the transition state, and tetramethylguanidine as the base component in chloroform at 50 °C (Scheme 13), products **27** were obtained in good isolated yields and enantioselectivities from moderate to good. Based on previous DFT studies on the intramolecular α-alkylation of aldehydes, the present intermolecular reaction was proposed to proceed through transition state **TS-6** in which the protonated aminocatalyst activates the leaving group departure through electrostatic stabilization of the developing negative charge at the halide, while further stabilization may operate through π−π interactions within both aryl moieties of the reactants.

**Scheme 14:**
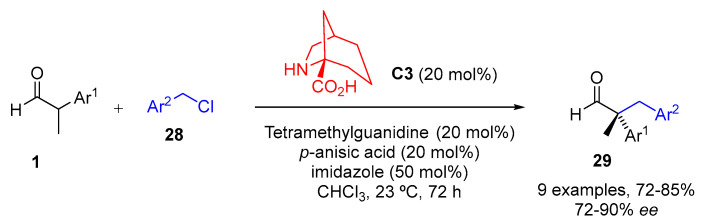
Optimizing conditions for the α-amino acid-catalyzed benzylic alkylation of branched aldehydes.

The above α-alkylation protocol requires relatively high catalyst loading (30 mol %), long reaction times (for 144 h at 50 °C), and the addition of large excesses of acid and base (5 equiv. each) as a buffer. Lee and coworkers described a modified α-benzylic alkylation of branched aldehyde with benzyl chlorides using the same cyclic secondary α-amino acid catalyst **C3** and imidazole as the key acid scavenger for success, along with substoichiometric amounts of a guanidine base and *p*-anisic acid (Scheme 14) [63]. Adducts **29** were thus obtained in good yields and high enantioselectivities. Curiously, the use of benzyl bromides instead of chlorides was detrimental for this catalytic reaction.

**Scheme 15:**
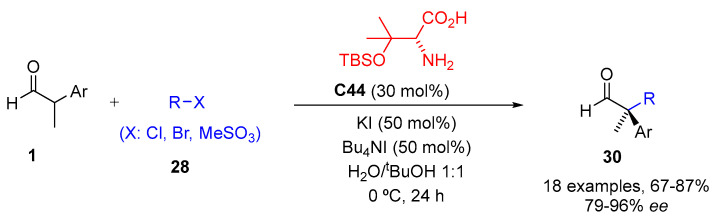
Aminocatalytic α-alkylation of branched aldehydes under phase transfer conditions.

Yoshida described the aminocatalytic α-alkylation of branched aldehydes under PTC conditions mainly using allylic and propargylic-type halides and sulfonates [64]. The reaction was promoted by 30 mol% of the serine-derived amino acid **C44** and required as cocatalysts substoichiometric amounts of KI and Bu_4_NI in a water/*tert*-butanol mixture (Scheme 15). The resulting adducts **30** were obtained in high yields and selectivities above 79% *ee*. The method appears to be limited to primary halides, whereas secondary halides led to unpractical yields.

(iii) 1,2- and 1,4-addition reactions

**Scheme 16:**
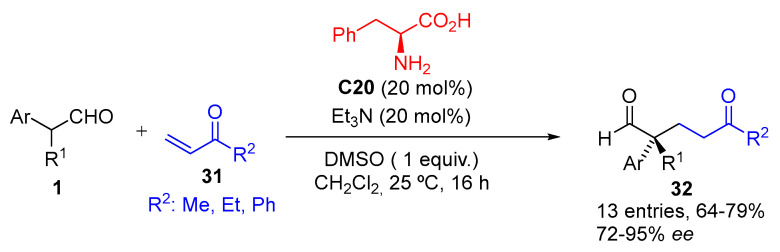
Enantioselective Michael reaction of α-branched aldehydes with enones.

Conjugate additions of aldehydes to Michael acceptors are a major C-C bond-forming reaction category within this area of catalysis [65,66,67,68,69,70,71]. Following up previous contributions in the area by the group, in 2015, Yoshida laboratory reported the addition of α-branched aldehydes to vinyl ketones **31** in the presence of 20 mol% of a chiral α-amino acid (e.g., phenylalanine **C20**) with the assistance of an accompanying base (Scheme 16) [72]. Various primary α-amino acids could promote the reaction, but phenylalanine gave the best balance of reactivity and enantioselectivity. Similarly, among the accompanying amine bases, tertiary amine bases like triethylamine or Hünig’s base were the best. The reaction was faster in polar solvents than in CH_2_Cl_2_, but the amount of highly polar byproducts increased. Addition of 1 equivalent of DMSO was beneficial for the conversion and yield without detrimental side reactions. The reaction was very sensitive to steric effects on the substrate, with the scope essentially limited to R^1^=Me groups, while β-substituted enones were not reactive enough under the present conditions. It is of remark that 2-phenyl butanal reacted with methyl vinyl ketone, providing the corresponding adduct in excellent 91% *ee* enantioselectivity, albeit only 39% conversion was achieved after 20 h.

**Scheme 17:**
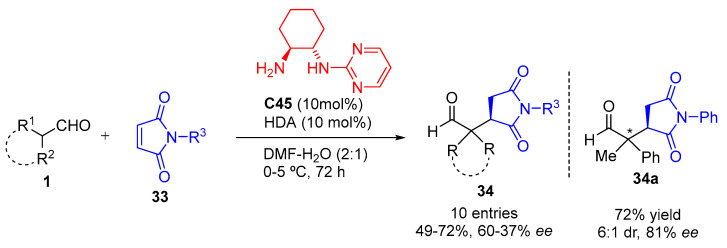
Aminocatalytic asymmetric addition of branched aldehydes to succinimides.

In that same year, Nájera and Gómez-Bengoa demonstrated that primary-secondary diamines derived from *trans*-cyclohenane-1,2-diamine may catalyze the conjugate addition of α-branched aldehydes to maleimides (Scheme 17) [73]. Using 10 mol% catalyst **C45** and hexanodioic acid (HDA, 10 mol%) as the cocatalyst in aqueous DMF at 10 °C, the corresponding succinimide adducts **34** were obtained in yields from moderate to good and variable enantioselectivities. Curiously intriguing, the sense of the maleimide face selectivity was solvent-dependent, affording as the major product the (*R*)-enantiomer in highly polar solvents (DMF-H_2_O), the (*S*)-product (although in low *ee*) in the nonpolar solvent dichloromethane, and a racemic mixture in toluene. Based on DFT calculations, this stereoswitch was rationalized in terms of the prevalence or not of H-bonding interactions between the maleimide carbonyl and the NH group of the aminopyrimidine catalyst in nonpolar and polar solvents, respectively. A single example using prostereogenic 2-phenylpropanal was reported to provide adduct **34a** in 72% yield, 6:1 dr, and 81% *ee* (configuration of the α-stereocenter not determined).

**Scheme 18:**
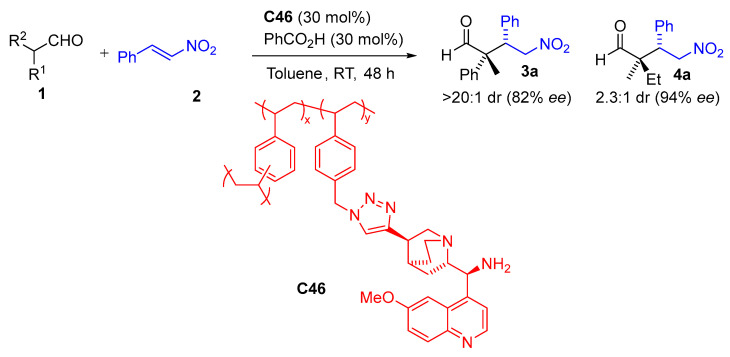
Polymer-supported chiral aminocatalysts for the enantioselective conjugate addition of branched aldehydes to nitrostyrenes.

Benaglia and coworkers developed a solid supported 9-amino *epi*quinine derivative **C46** as a polymeric aminocatalyst to promote the Michael addition of aldehydes, and specifically α-branched aldehydes, to β-nitrostyrene under flow conditions (Scheme 18) [74]. Preparation of the catalyst was accomplished through radical polymerization of a triazole-linked 9-amino *epi*quinine-styrene hybrid, which could be obtained by a Cu-catalyzed click cycloaddition between the corresponding azide-functionalized styrene and acetylenic 9-amino *epi*quinine derivative. Using benzoic acid as an additive, thus-formed catalyst **C46** was able to promote the addition of α-branched aldehydes to nitrostyrene with good yields and generally good selectivity. In particular, the prochiral α-phenylpropanal and α-methylbutanal reacted with nitrostyrene to yield the corresponding adducts **3a** and **4a** in >20:1 and 70:30 diastereoselectivities, and of 82% and 95% *ee*, respectively. Curiously, as products structures in Scheme 18 show, there was no apparent correlation between the products’ configuration and the relative size of the α-substituents. The supported catalyst could be reused in successive runs, although second and ulterior runs gave variable yields and selectivities. Catalyst reusability could be improved upon washing the catalyst-containing column with a solution of benzoic acid before each use.

**Scheme 19:**
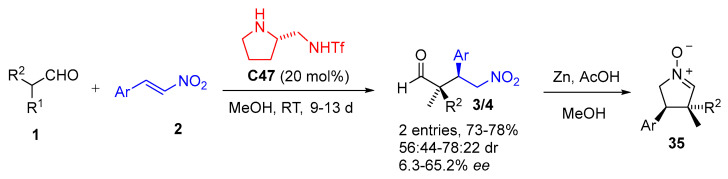
A secondary amine-catalyzed asymmetric addition of branched aldehydes to nitrostyrenes.

The Szcześniak group applied the pyrrolidine-sulfonamide catalyst **C47**, previously developed by Wang [75], to the addition of α-branched aldehydes to β-nitrostyrenes (Scheme 19) [76]. The majority of examples deal with aldehydes bearing two identical α-substituents. Only two entries with unequal substituents (R^1^: Me; R^2^: Et, Ph) were reported, leading to adducts **3**/**4** in poor dr (R^2^: Et, 56:44 dr) or low enantioselectivity (R^2^: Ph, 6.3% *ee*). These adducts were used as an entry to cyclic nitrones under the treatment with Zn/AcOH/MeOH.

**Scheme 20:**
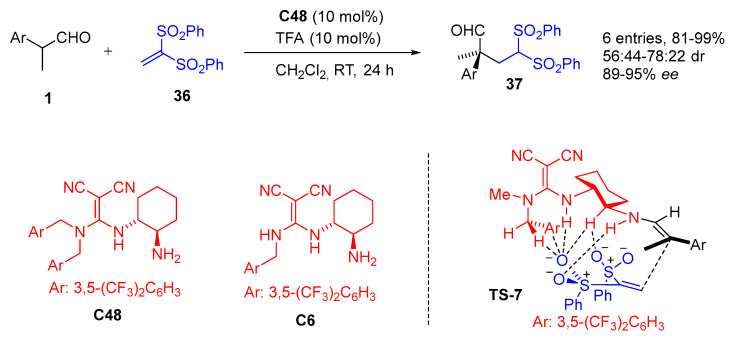
Asymmetric conjugate addition of branched aldehydes to vinyl bis-sulfone.

More recently, the Yanai and Miura group described the enantioselective Michael addition of α-aryl propanaldehydes to vinyliden bis(sulfones) promoted by their diaminomethylenmalononitrile/primary amine catalyst **C48** (Scheme 20) [77]. Using trifluoroacetic acid as an additive in dichloromethane as the solvent, excellent enantioselectivities of the respective α-quaternary aldehyde adducts were obtained under smooth conditions (RT, 24 h). The unexpected finding was that the *N,N*-disubstituted catalyst **C48**, which contains a single *NH* group available for H-bonding interactions, was more effective than the *N*-monosubstituted analog **C6**. Control experiments using different batches of catalysts with variable enantiopurities confirmed a linear relationship between catalyst and products *ee,* thus indicating that a single catalyst molecule intervenes in the stereochemistry-determining step. Based on DFT calculations, the authors proposed a transition state **TS-7,** in which the bifunctional activation of donor and acceptor components through an H-bonding network operates.

**Scheme 21:**
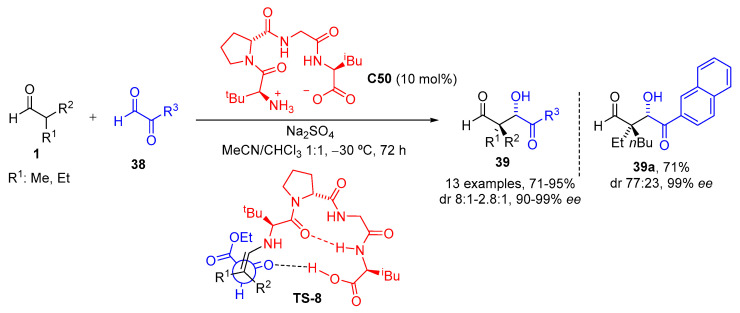
A tripeptide-catalyzed asymmetric crossed-aldol reaction of branched aldehydes with glyoxylates.

Da and coworkers demonstrated that β-turn tetrapeptides with free *N*- and *C*-terminus (**C50**) were able to catalyze the asymmetric aldol reaction of α-branched aldehydes and α-carbonyl aldehydes, i.e., glyoxylates and α-ketoaldehydes, to biomimetically synthesize acyclic all-carbon quaternary center-bearing 1,4-dicarbonyls in high yield and excellent enantioselectivity under mild conditions (Scheme 21) [78]. The high enantioselectivity observed is ascribed to the spatially restricted environment imposed by the tetrapeptide on the resulting enamine intermediate, as shown in **TS-8**. It is quite remarkable the high enantioselectivity was achieved even with the challenging 2-ethyl hexanal to afford aldol adduct **39a**.The practicality of this protocol was illustrated in an enantioselective synthesis of (*R*)-pantolactone, the key intermediate of vitamin B5 synthesis.

## 3. Methods Based on Brønsted Base Activation

**Scheme 22:**
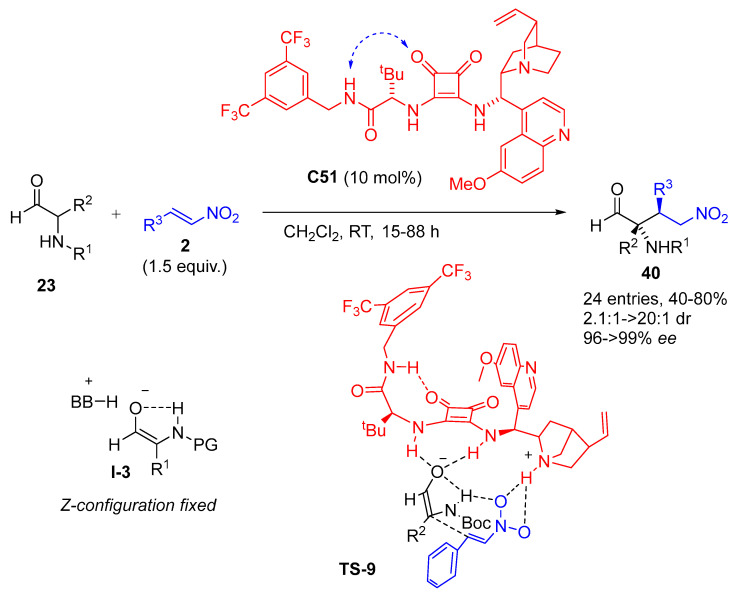
Bifunctional Brønsted base/H-bonding catalyst activation approach for Michael additions of α-amino-aldehydes.

Addition reactions of suitable pronucleophiles to unsaturated electrophilic reagents may be greatly accelerated by a bifunctional Brønsted base/H-bonding catalyst involving proton shuttle as the key event, and have delivered many examples in asymmetric catalysis [79,80,81,82,83]. Despite the simplicity of the approach, until recently, forging highly enantioselective carbon–carbon bond-forming reactions of enolizable aldehydes based on this activation approach has remained elusive. Besides the potential formation of self-aldolization byproducts, control of the reaction stereochemistry is challenging owing to the possible formation of mixtures of *E* and *Z*-enolate intermediates. In addition, the lack of covalent interactions between the substrate and the catalysts may lead to highly flexible transition states, and thus suboptimum chirality transfer. In 2021, Palomo and Mielgo described asymmetric addition reactions of α-amino branched aldehydes to nitroolefins based on a bifunctional Brønsted base/H-bonding catalyst activation approach (Scheme 22) [84]. Authors hypothesized that the internal H-bond in the α-amino aldehyde should fix the intermediate enolate geometry to *Z*, thus facilitating efficient enantiofacial discrimination during a key C-C bond formation step. Among the various bifunctional catalysts tested, **C51**, bearing multiple H-bond donor sites, proved to be superior in terms of reactivity and stereoselectivity. The method provided stereoselective construction of α-amino aldehydes and derivatives, therefrom featuring two vicinal quaternary and tertiary carbon stereocenters. The aldehyde substrate scope ranges from α-methyl to larger α-alkyl α-amino aldehydes. On the other hand, not only *N-*Cbz or *N-*Boc derivatives, but other simple amides were also tolerated as *N*-protecting groups. A DFT-based study of the key C-C bond forming step confirmed participation of *Z*-configured enolate intermediate **I-3** and unveiled the internal H-bond within the catalyst amide NH and squaramide C=O groups as key activation and rigidification elements. Effective steric shielding of one face of the enolate by the bulky *tert*-butyl group of the catalyst, as in **TS-9**, would explain the observed sense of asymmetric induction and the high stereoselectivity.

**Scheme 23:**
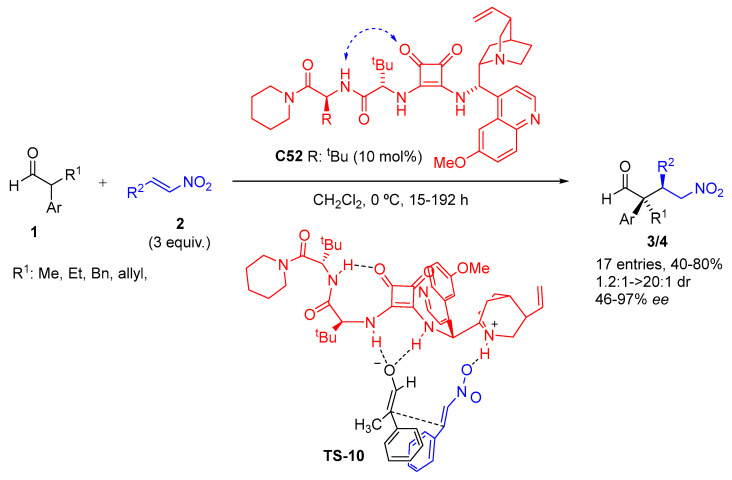
Extension of the above catalytic addition to α-alkyl α-aryl aldehydes.

In subsequent work, the same group demonstrated that this activation approach is also applicable to α-alkyl,aryl branched aldehydes to afford adducts **3/4** bearing all-carbon quaternary-tertiary vicinal carbons in good yields and usually high stereoselectivity (Scheme 23) [85]. Unlike in most other catalytic methodologies for direct enantioselective α-functionalization of branched aldehydes, aldehydes with larger alkyl chains at Cα, such as ethyl, benzyl, or allyl, were competent substrates for this reaction, although the accompanying diastereo- and enantioselectivities were comparatively lower, while reaction times increased considerably. After screening several bifunctional BB/HB catalysts, the researchers found the squaric acid-derived amino acid peptide catalyst **C52** performed slightly better than **C51**. These highly tunable catalysts incorporate in their structure an additional amino acid unit that allows for further optimization of catalyst activity and selectivity. According to computational modeling of the catalytic reaction, with α-alkyl,aryl-branched aldehydes, the most stable transition state **TS-10** involves an *E*-configured enolate intermediate, which reacts through its *Si* face. The observed facial selection was once again consistent with the existence of an intramolecular H-bonding interaction between the NH of one of the *tert*-leucines and the carbonyl of squaramide moiety of the catalyst that fixes the catalyst conformation independently of the activation mode considered.

## 4. Methods Based on Metal-Centered Activation Catalysis

**Scheme 24:**
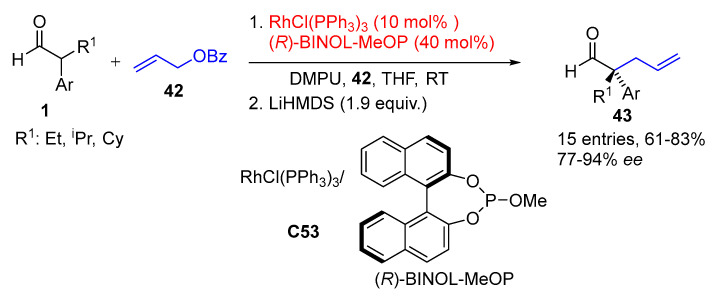
Rhodium-phosphite catalyzed asymmetric allylic alkylation of α-branched aldehydes.

Catalytic methods for the enantioselective α-functionalization of α-branched aldehydes that proceed through the corresponding metal enolate have been rarely reported. Even racemic versions of the approach have not been developed to a large extent. For instance, Sauthier and coworkers [86] developed a racemic α-allylic alkylation of branched aldehydes with allylic alcohols, while the Mazet group described a racemic Pd-catalyzed benzylation of branched aldehydes using benzyl methyl carbonates [87]. One general problem is that metal enolates tend to be highly nucleophilic; hence, aldehyde self-aldolization represents a competitive undesired process. Regarding asymmetric versions development, controlling the *E/Z* geometry of the formed metal enolate intermediate is another challenge for attaining practical levels of stereoselectivity during subsequent electrophile approaching. In 2016, the Evans group reported a rhodium-phosphite catalyzed LiHMDS/DMPU system-promoted asymmetric allylic alkylation of α-branched aldehydes with allyl benzoate **42** (Scheme 24) [88]. The order of addition of reagents is important, with LiHMDS (1.9 equiv. with respect to the limiting reagent **42**) being added last and dropwise over a period of 30 min. A salient feature of the method is that aldehydes with alkyl groups larger than methyl, such as ethyl or even branched isopropyl and cyclohexyl, were tolerated, affording the corresponding adducts **43** in high selectivity. Control experiments in which the lithium enolate intermediate with stereodefined *E* (*E/Z* 96:4) or *Z* (*E/Z* 7:93) configuration was generated independently from the respective silyl enol ether by treatment with MeLi, afforded, in the presence of the Rh catalyst **C53**, product **43** of the same configuration, but slightly lower enantioselectivity (71% and 68% *ee*, respectively). These observations, along with the fact that both *E* and *Z* configured metal enolates are observed in solution, suggest that dynamic kinetic resolution of enolates might occur. Authors also proved that the silylamide base plays a crucial role to assure high selectivity.

**Scheme 25:**
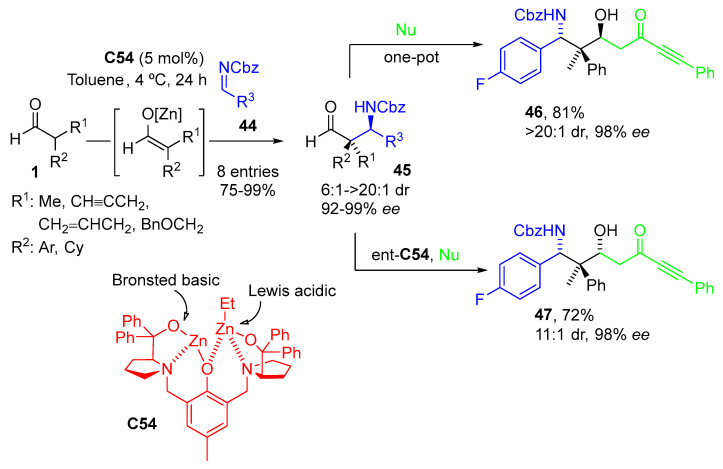
Dinuclear zinc-aminoalcohol catalyzed Mannich addition reactions.

A relevant asymmetric variant that does not require a stoichiometric strong base for enolization has been developed by Trost based on his dinuclear zinc-aminoalcohol catalyst **C54**, which is obtained through the coupling of ZnEt_2_ and phenol-tethered diphenylprolinol ligand (Scheme 25) [89]. The resulting bifunctional catalyst displaying sites with Brønsted basic and Lewis acidic character is able to promote the Mannich addition reaction of α-branched prochiral aldehydes **1** and *N*-carbamoyl imines **44** derived from aromatic aldehydes to afford adducts **45** in very high yields and stereoselectivities. Instances of reactions run at 2 mmol scale with catalyst loading as low as 1 mol% were also reported. This approach was a priori counterintuitive given that, previously, branched aldehydes were effectively employed as electrophiles for Zn-ProPhenol catalyzed reactions [90]. Apparently, the imine substrates capable of two-point binding with the metal catalyst are superior electrophiles compared with branched aldehydes, thus preventing self-aldolization. Of note, thus obtained adducts could be further elaborated by the addition of various nucleophiles, leading to densely functionalized products **46** and **47** in a stereodivergent manner. The stereochemical course of the second sequential step is governed, in one case, by the chiral Mannich substrate **45** in one-pot experimental procedures, while access to the complementary diastereomer **47** can be achieved in a two-step protocol involving catalyst interchange (filtration of reaction mixture and addition of ent-**C54**) and addition of the nucleophile.

**Scheme 26:**
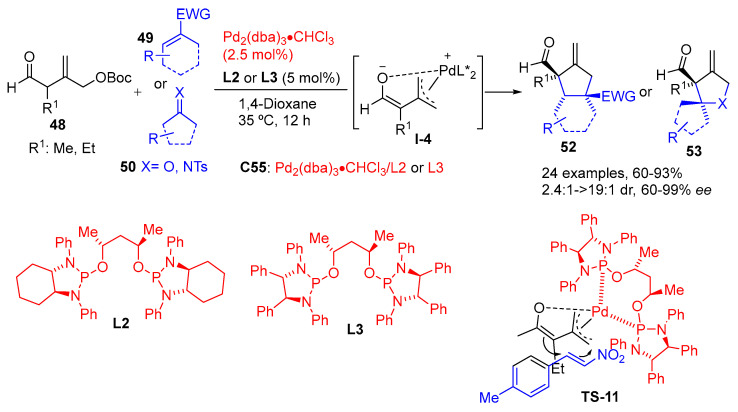
Pd-catalyzed [3+2] coupling as a general approach for preparation of chiral α-quaternary aldehydes.

Trost and coworkers have developed a novel strategy to control the enantioselective construction of chiral α-quaternary aldehydes from racemic α-branched aldehydes. Aldehydes with a tethered allyl carbonate moiety **48** upon treatment with the palladium catalyst **C55** would evolve into a chiral π-allyl-Pd complex neighboring the enolate (**I-4**). Formal [3+2] coupling of this intermediate with the acceptor reaction partner, e.g., nitroolefins, ketones, and imines, then provides the corresponding cyclization adducts **52**/**53** with up to three new stereocenters in good yields and usually high enantio- and diastereoselectivity (Scheme 26) [91]. A palladium-centered highly ordered transition state model **TS-11** was proposed to explain the stereochemical course of the reaction observed.

## 5. Methods Based on Dual Activation Involving Amine and Metal Co-Catalysis

After initial development of aminocatalytic methods for the direct α-functionalization of α-branched aldehydes via enamines, it was eventually found that dual catalysis by combination of an (usually primary) amine catalyst with a second independent cocatalyst presents new opportunities for improving the efficiency of the catalytic reactions and ultimately expanding the reactivity scope to new applications. The use of two discrete catalytic entities in the reaction allows for independent optimization of each catalyst component. Moreover, either catalyst or both may be chiral; thus, up to four different stereochemical combinations of the two cocatalysts are plausible, which not only provides more room for fine-tuning of the reaction stereoselectivity, but also the implementation of fully stereocontrolled routes to all possible stereoisomers of the products became a realistic goal. While these are potential advantages of dual amine/metal catalysis, an important constrain of the approach is that many metal complexes are not compatible with amines and therefore catalyst self-quenching is a potential risk that needs to be considered.

**Scheme 27:**
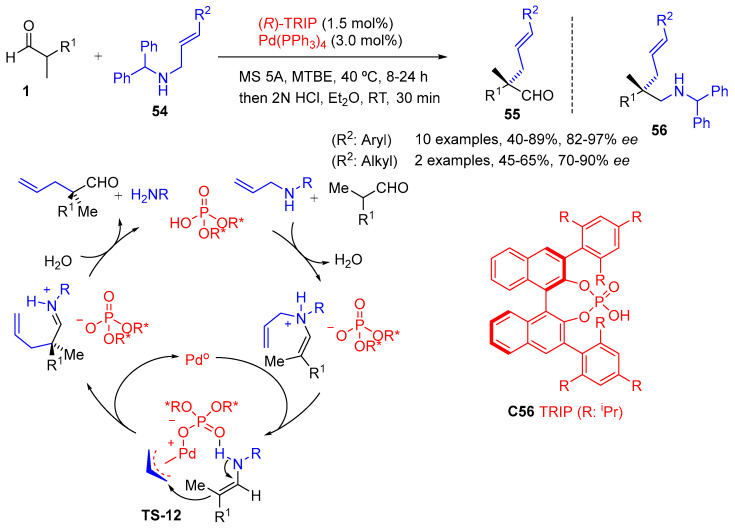
Chiral organophosphoric acid (*R*)-TRIP (**C56**) and Pd(PPh_3_)_4_ as the cocatalyst for α-allylation of aldehydes.

In pioneering work in the area, in 2007, List documented [92] the reaction between secondary allylamines and α-branched propionaldehydes to afford the corresponding all-carbon α-quaternary aldehydes in the presence of a chiral organophosphoric acid (*R*)-TRIP (**C56**) and Pd(PPh_3_)_4_ as the cocatalyst (Scheme 27). In this ingenious conception, after initial amine-aldehyde condensation, the resulting enamine would undergo an acid-promoted fragmentation to provide a cationic Pd-coordinated allylic species and a nucleophilic NH-enamine. Both species would then collapse with the formation of the new C-C bond under the influence of the chiral phosphate anion that may act as either a chiral counterion species or chiral ligand for palladium, or both. The reaction investigation led to identify *N*-benzhydryl allyl amines as best suited allylic amines and TRIP as the optimum organophosphoric acid catalysts to construct quaternary products **55** with generally good yield and enantioselectivity. Compared to the α-aryl propionaldehydes, the parent α-alkyl derivatives proved to be more challenging substrates and afforded adducts with diminished enantioselectivity. Since the initial reaction product is the corresponding imine as confirmed by GC-MS, the reaction work-up with NaBH_4_ instead of acidic hydrolysis rendered the corresponding *N*-benzhydryl amines, e.g., **56**.

**Scheme 28:**
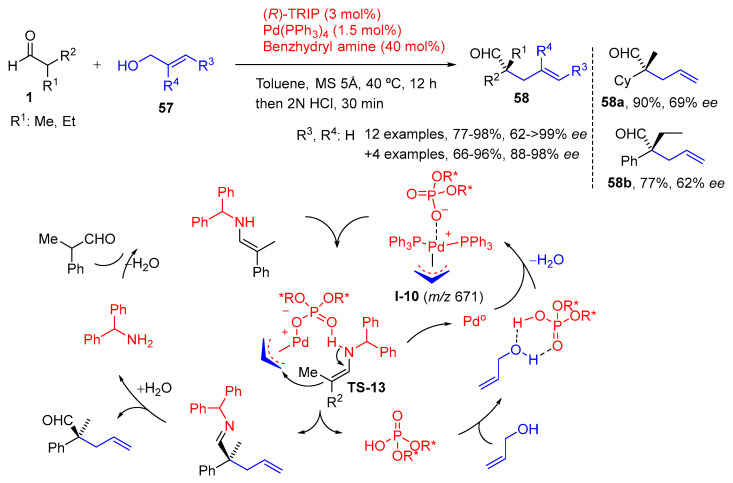
Three different catalytic species approaches for the asymmetric allylation of aldehydes.

While the above method cannot be characterized as truly aminocatalytic, that demonstration paved the way to implement genuine aminocatalytic dual catalysis approaches. In a variation of the method, List and coworkers used simple allylic alcohols **57** as the electrophilic π-allyl-Pd precursors by the concerted action of three different catalytic species, [Pd(PPh_3_)_4_], benzhydryl amine, and TRIP (Scheme 28) [93]. The reaction in the presence of Pd complex and TRIP only, but in the absence of benzhydryl amine, also proceeded, although afforded products **58** with poor enantioselectivity. The key role played by the amine was rationalized assuming that *E-*enamine would form predominantly under optimal conditions, ensuring high enantioselectivity through **TS-13**, while the background enol-mediated reaction would presumably proceed via a mixture of *E/Z* enol intermediates instead. α-Aryl propionaldehydes afforded products **58** in excellent yields (94–98%) and enantioselectivity (88 −> 99% *ee*). In contrast, the more challenging 2-cyclohexylpropanal and 2-phenyl butanal also reacted with allylic alcohol, but with poorer enantioselectivity (69% and 62% *ee*, respectively).

**Scheme 29:**
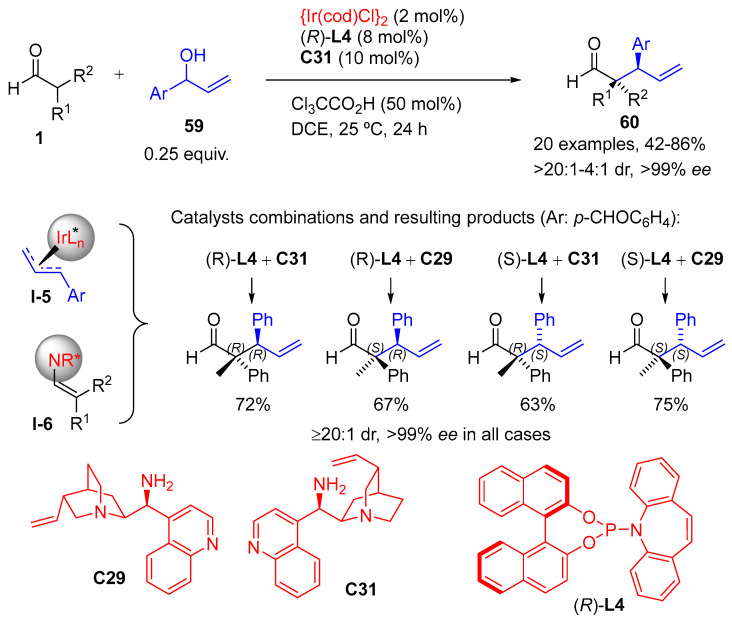
Dual catalysis using chiral primary amine/iridium-phosphoramidite organometallic complexes for the asymmetric allylation of aldehydes.

In an elegant study, Carreira disclosed an access to the full matrix of stereoisomeric products resulting from the allylic α-alkylation reaction of branched aldehydes with allylic alcohols under dual catalysis effected by a chiral primary amine and a chiral iridium-phosphoramidite organometallic complex (Scheme 29) [94,95,96]. Based on the observed stereochemical outcomes, it could be concluded that while the primary amine catalysts **C29**/**C31** have full control over the newly formed α-stereocenter (local stereoinduction on prochiral enamine intermediate), the configuration of the ligand **L4** on the iridium complex fully determines the configuration of the β-stereocenter (local stereoinduction on prochiral allylic cation-metal complex). This way, each pair of co-catalysts’ combinations induces almost exclusive formation of one isomeric product out of the four possible, with virtually complete enantiocontrol. Remarkably, the reaction tolerated a wide range of aryl vinyl carbinols **59** and branched aldehydes **1** of various types, including α,α-dialkyl aldehydes.

**Scheme 30:**
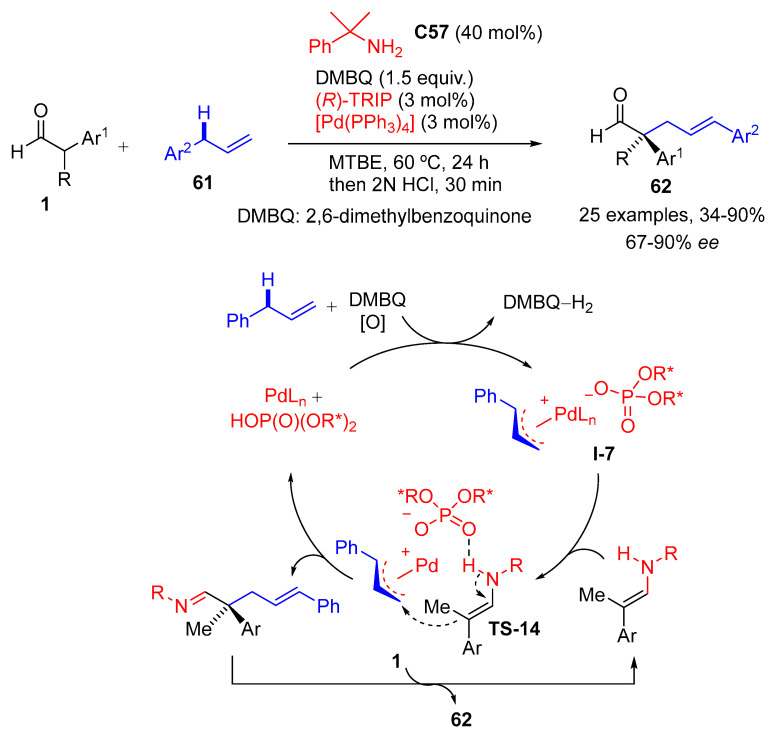
Asymmetric counterion catalysis in combination with palladium-catalyzed allylic C-H activation.

In 2014, Gong et al. described the first enantioselective α-allylic alkylation of branched aldehydes with nonfunctionalized alkenes by combining asymmetric counterion catalysis and palladium-catalyzed allylic C-H activation (Scheme 30) [97]. The method tolerates a broad range of (mainly) α-aryl propanaldehydes and terminal alkenes **61** and affords products **62** with good yield and high enantioselectivity for most cases. The underlining concept is that in the presence of palladium(0), an oxidant such as DMBQ and a chiral phosphoric acid such as TRIP, olefins bearing allylic C-H bonds could be oxidized to generate the π-allyl palladium phosphate complex **I-7** as critical intermediate for stereoselective allylic alkylation of in situ formed enamine (**TS-14**), as demonstrated previously by List. Terminal alkenes **61** bearing aryl and alkenyl substituents at the allylic position were equally suitable. However, the reactivity appeared to be greatly influenced by the aryl substituent. Thus, allylbenzenes with an electron-deficient or alkyl substituent at *para* proceeded smoothly, while with strongly electron-donating or a chloro substituent at *para* were less reactive and required higher temperatures (80 °C) to ensure good conversion.

**Scheme 31:**
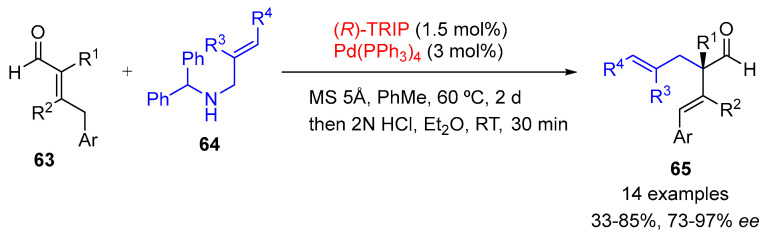
Deconjugative α-allylic alkylation reaction of α,β-unsaturated aldehydes.

Very recently, Brenner-Moyer demonstrated that under conditions developed by List, conjugated enals may undergo a deconjugative α-allylic alkylation reaction with *N*-allylic *N*-benzhydryl amines **64** in the presence of both TRIP and Pd(PPh_3_)_4_ cocatalysts (Scheme 31) [98]. Under optimized conditions, α-methyl enals **63** (R^1^: Me) afforded the α-allylated products **65** in good yields and very good enantioselectivity. The reaction involving an α-ethyl enal **63** (R^1^: Et) required a modified TRIP catalyst to proceed satisfactorily. While the deconjugated α-allylated adducts **65** were the main or exclusive products, appreciable amounts of the γ-functionalization conjugated enal were also isolated when enals bearing electron-deficient aryl groups (Ar-CN, Ar-NO_2_, and Ar-CF_3_) were employed.

**Scheme 32:**
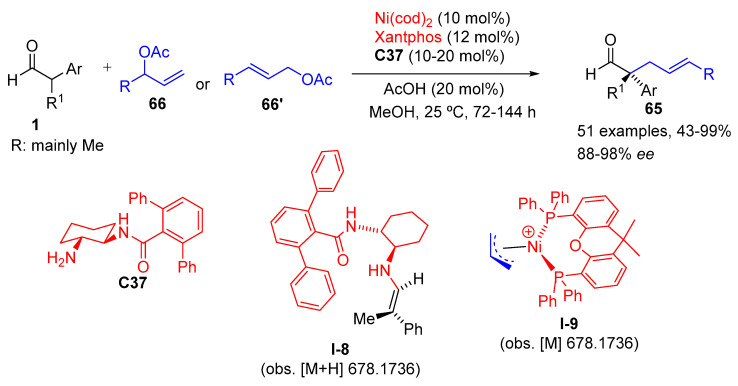
Nickel/enamine cooperative dual catalysis for highly enantioselective allylic α-alkylation of branched aldehydes.

Complementing the above methods that rely on palladium catalysis, recently, Shen reported nickel/enamine cooperative dual catalysis for triggering the highly enantioselective allylic α-alkylation of branched aldehydes with allyl acetates used as alkylating agents (Scheme 32) [99]. The method, which requires nickel cyclooctadiene and Xantphos as the optimal ligand, on the one hand, and primary amine/amide **C37**, led to linear vinyl adducts **65,** regardless of the linear or branched nature of the allyl ester, and tolerated a wide variety of substituents on both the aldehyde α-position and the allylic acetate. Participation of both the intermediate enamine **I-8** generated from coupling of the aldehyde with the chiral bifunctional primary amine/amide **C37** and the nickel-Xantphos complex **I-9** was supported by detection of both species by high resolution mass spectroscopy in the solution. The method was demonstrated to be widely applicable to a variety of aldehydes and allylic esters, and its synthetic potential was illustrated by achieving short syntheses of bioactive compounds (+)-eptazocine, (-)-aphamorphine, and antagonists of ent-5HT1A and NK-3 receptors, respectively.

**Scheme 33:**
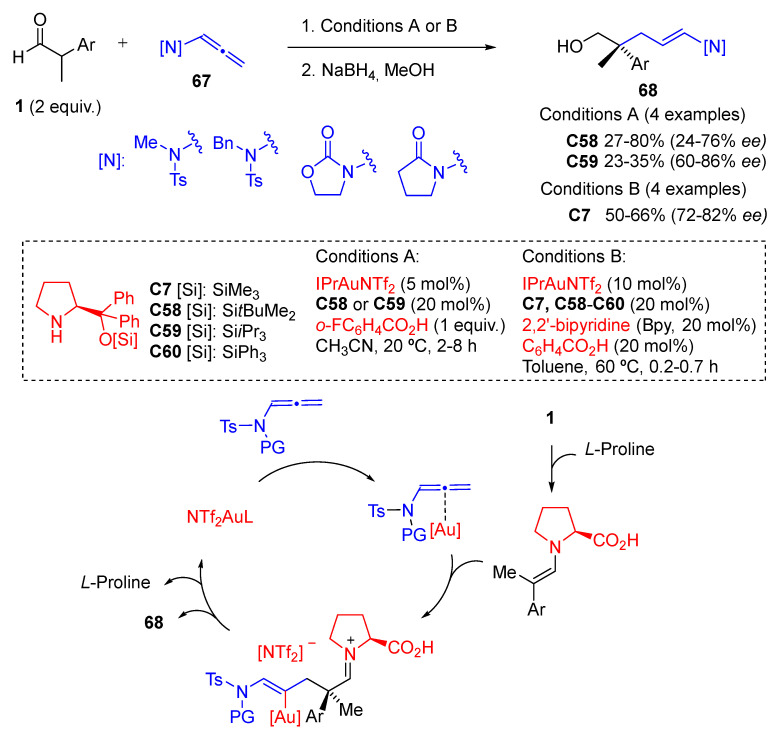
Enamine-mediated α-allylation reaction of branched aldehydes with allenamides.

Besides allylic alcohols and esters, allenes constitute another class of electrophilic allylic alkylating agents upon activation with certain transition metal complexes. Combinations of allenic substrates with Pd, Au, or Cu-based catalysts have been applied as electrophilic reagents in enamine-mediated α-functionalization reactions of branched aldehydes. Previously, Dixon and coworkers had reported intramolecular α-allylic alkylation of linear aldehydes bearing a tethered allene functionality (allenamides carbocyclization) using an amine catalyst and a palladium cocatalyst [100]. Later on, in back-to-back papers from the group of González, on the one hand, and of Mascareñas and López, on the other hand, reported independently the intermolecular version in which an enamine-mediated α-allylation reaction of branched aldehydes using allenamides and gold(I) co-catalysis was achieved. In both Au-catalyzed procedures, allenamides with different *N*-acyl and *N*-sulfonyl groups attached were employed as reaction partners, and the corresponding *N*-functionalized α-allylic quaternary aldehydes were obtained with *E*-configuration at the enamide terminus (Scheme 33). Conditions developed by the Gonzalez group consist of proline as the aminocatalyst and IPrAuN(Tf)_2_ as the metallic co-catalyst in the presence of one equivalent of 2-fluorobenzoic acid in acetonitrile at 20 °C [101]. Products were isolated in variable yields as the alcohols **68** upon reduction with NaBH_4_. Enantioselective versions for branched aldehydes were carried out using diphenylprolinol silyl ethers as aminocatalysts instead of proline (Conditions A). On the other hand, Mascareñas and López identified slightly different conditions (Conditions B) in which a lesser quantity of the benzoic acid was sufficient, but the use of a diamine additive, preferably Bpy, and heating conditions in toluene were necessary for obtaining the best results [102]. In both studies, diarylprolinols with various protecting silyl groups were screened as the aminocatalyst. Under conditions A, the TIPS-masked prolinol **C59** afforded better stereocontrol than SitBuMe_2_ ether **C58**, but worse yields. In turn, under conditions B, the catalyst bearing the smallest silyl protecting group (SiMe_3_) was the most active, while the catalyst with bulkier silyl groups (SitBuMe_2_, SiEt_3_, and SiPh_3_) afforded higher enantioselectivities in some instances, at the expense of lower yields or longer reaction times. It is worth noting that the bis(trifluoromethyl)phenyl prolinol ethers (e.g., **C8**) were totally ineffective under the above conditions.

**Scheme 34:**
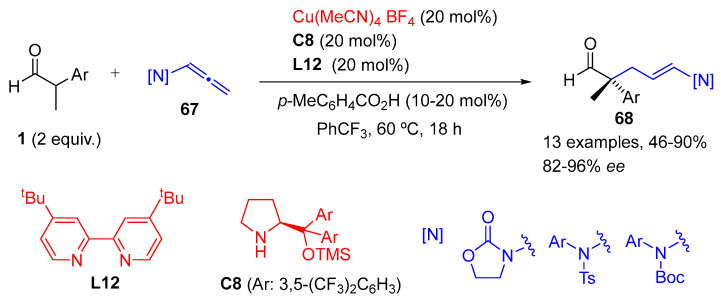
Asymmetric allylation using Cu(I) complex as the metallic cocatalyst with bis(3,5-trifluoromethylphenyl) prolinol.

In a variation of the above methods involving allenamides **67**, the group of Taillefer and Monnier have found that when a Cu(I) complex is used as the metallic cocatalyst, bis(3,5-trifluoromethylphenyl) prolinol TMS ether **C8** performs superior to related aminocatalysts for addition reactions carried out at 70 °C (Scheme 34) [103]. This reaction afforded compounds **68** in good regio- and stereoselectivity, with the linear product formed exclusively as an *E*-configured isomer. After the screening of solvents, additives, and conditions, it was found that trifluorotoluene as the solvent and a combination of chelating 2,2′-bi(tert-butylpyridine) **L12** ligand for copper and *p*-toluic acid as a weak acid additive were the optimum. Various electron-withdrawing nitrogen-containing groups, such as oxazolidinones, sulfonyl amide, and Boc-amide, were equally compatible as allenamide reagents in this development.

**Scheme 35:**
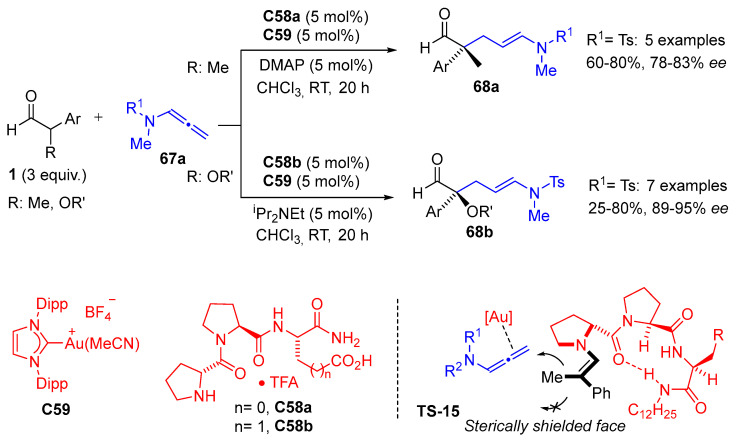
Dual catalysis approach to the α-alkylation of branched aldehydes using a proline-based tripeptide as the catalyst.

Very recently, Wennemers has described a dual catalysis approach to the α-alkylation of branched aldehydes with allenamides, in which a proline-based H-_D_Pro-Pro-Glu-NH_2_ **C58a** or H-_D_Pro-Pro-Asp-NH_2_ tripeptide **C58b** is employed as the key amine catalyst (Scheme 35) [104]. Among various metal complexes known to activate allenamides, gold complexes **C59** were identified as suitable, whilst other complexes of Cu, Pd, Rh, and Ir showed to be inactive or gave poor yields. The reaction tolerated well both α-aryl propionaldehydes and α-alkoxy phenylacetaldehydes. For the former subset of aldehydes, the best results were obtained with salt **C58a** as a catalyst and DMAP as a base, while salt **C58b** and Hünig’s base [105] performed better for the latter. With respect to allenamide scope, the *N*-Ts allenamides were best suited, while the *N*-Boc and *N*-alloc allenes led to diminished yields, and allenamides with a γ-substituent (Me) resulted unreactive. Control experiments, in combination with NMR data and X-ray analysis, proved that both additives, the acid and the amine base, along with the intramolecular salt bridge within the structure of these tripeptides, are crucial to help to separate the peptide and gold catalysts and disfavor non-productive interactions. The stereochemical outcome of the reaction is consistent with the gold-activated allenamide approaching the less sterically hindered face of the enamine, as shown in the model **TS-15**.

**Scheme 36:**
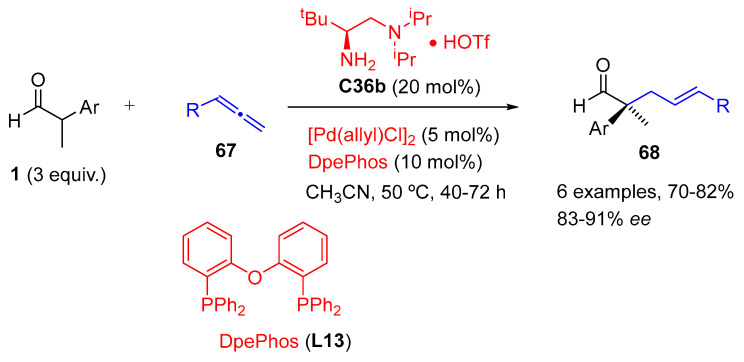
Dual activation approach using a primary amine chiral catalyst and a palladium complex co-catalyst for the coupling with allenes.

Based on the same dual activation approach, but using a primary amine chiral catalyst and a palladium complex as the co-catalyst, Luo and coworkers developed a highly enantioselective α-alkylation of α-aryl propionaldehydes extensible to alkyl- and aryl allenes (Scheme 36) [106]. After screening various tertiary-primary 1,2-diamines derived from *tert*-leucine, in combination with an equimolar quantity of triflic acid, the *N*-diisopropyl derivative **36b** was identified as the best performing aminocatalyst. In conjunction with a palladium-DpePhos complex for the activation of the allenic component, the addition of α-aryl α-methyl acetaldehydes **1** to aryl- and alkyl-allenes **67** proceeded in acetonitrile at 50 °C to afford *E*-olefins **68** in good yields and in enantioselectivities up to 91% *ee*.

**Scheme 37:**
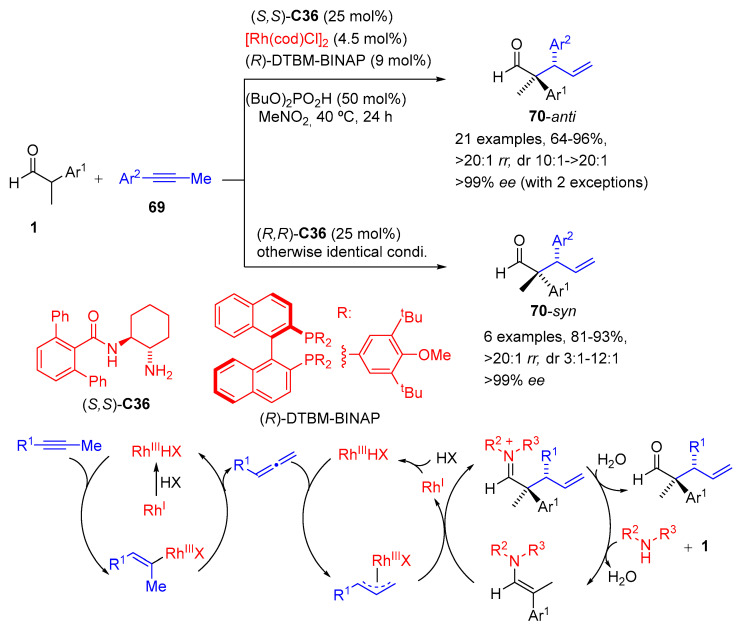
Dual catalysis protocol for reactions of branched aldehydes with aryl-methyl alkynes using rhodium hydride-catalyzed hydrometallation.

Dong et al. described a dual catalysis protocol to react intermolecularly α-aryl propanaldehydes and internal aryl-methyl alkynes, the latter activated through rhodium hydride-catalyzed hydrometallation (Scheme 37) [107]. Interception of the enamine intermediate with an electrophilic π-allyl complex produces α-allylated aldehydes with perfect regioselectivity in contrast to previous studies using metalorganocatalysts, in which intramolecular alkyne coupling gave α-vinylic aldehyde products. Various primary amines were proved to be effective catalysts for this transformation, but the Jacobsen’s catalyst **C36**, derived from cyclohexyl-1,2-diamine, provided the highest stereoselectivity in combination with the Rh-(*R*)-DTBM-BINAP cocatalyst. An interesting aspect of this development is that either *syn* or *anti* diastereomer of **70** may be selectively produced by using, respectively, (*R,R*)-**C36** or (S,S)-**C36** in combination with a (*R*)-configured DTBM-BINAP ligand on Rh. Thus, access to all possible stereoisomers by changing the handedness of each catalyst is possible. While a relatively large amount of amine catalyst is necessary (25 mol%), high diasteroselectivities, especially for *syn* isomer, and essentially perfect enantioselectivities were obtained. Three independent catalytic cycles are believed to operate in cascade as depicted in the mechanistic proposal of Scheme 37.

**Scheme 38:**
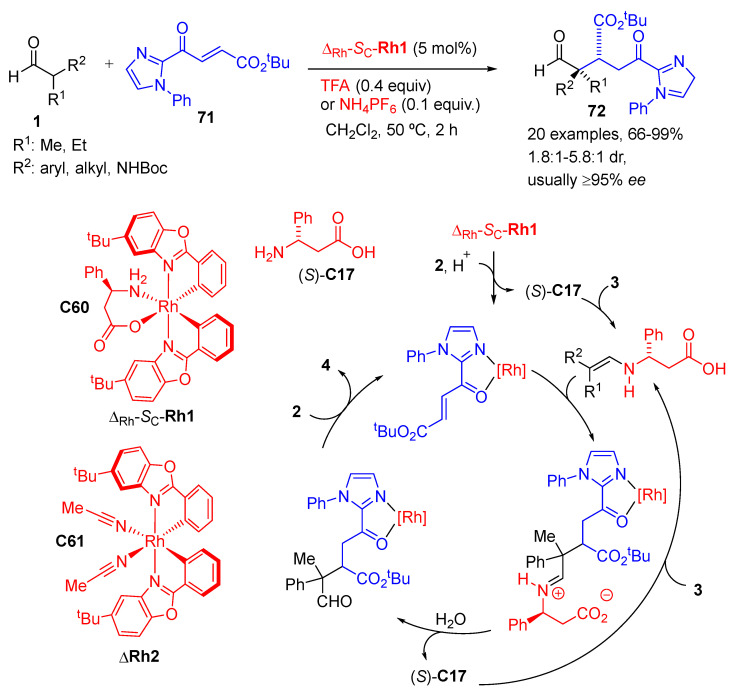
Complex dissociation renders two independent catalysts for Michael additions of branched aldehydes.

In this and other methods based on dual catalysis, the two co-catalysts are added to the reaction mixture as independent species that work in concert during the key stereo-determining step. In a different approach, Meggers demonstrated that the two active co-catalysts needed in the conjugate addition of aldehydes to an active Michael acceptor may be delivered in situ from the dissociation of a metal complex precatalyst. Thus, the complex Δ_Rh_-*S*_C_-**Rh1**, which is easily prepared as essentially pure diastereomer from the complexation of RhCl_3_ salt and β-phenylalanine and 5-*tert*butyl-2-phenylbenzoxazole as ligands, is able to catalyze the addition of branched aldehydes, including 2-phenyl-butanal and a variety of α-substituted propanals, to α,β-unsaturated 2-acyl imidazoles **71** in the presence of substoichiometric TFA (or NH_4_PF_6_) to afford adducts **72** in high yields (Scheme 38) [108]. The vicinal tertiary/quaternary stereocenters are generated with diastereoselectivities from moderate to good and very high enantioselectivity for the major isomer. The presence of TFA as an additive was important to shorten the reaction time (2 h at 50 °C) while maintaining the high stereoselectivity. On the other hand, the need for both cocatalysts for the reaction to proceed with high efficiency and selectivity was clear, as reactions run in the presence of phenylalanine ((*S*)-**C17**) alone or the catalyst **C60** alone proceeded sluggishly. A mechanism was proposed in which the TFA accelerates the release of phenylalanine from the complex by protonation.

## 6. Methods Based on the Merging of Enamine Activation and Photoactivation

**Scheme 39:**
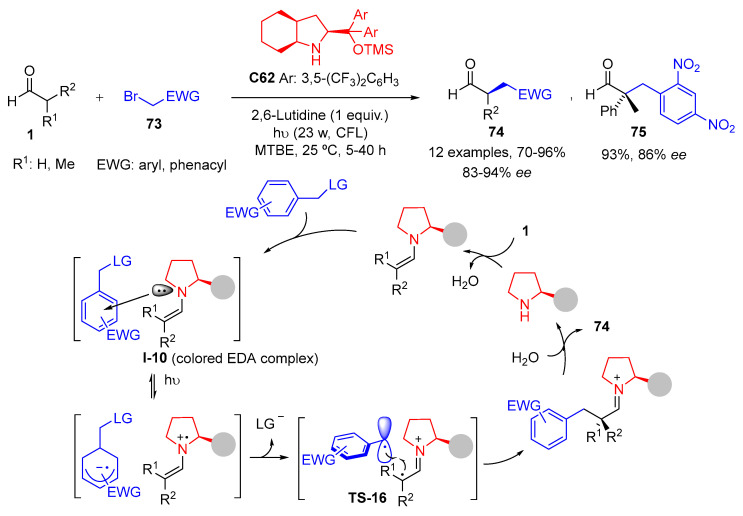
Photochemically driven aminocatalytic asymmetric α-alkylation of aldehydes with reactive alkyl bromides.

In 2013, Melchiorre et al. described a photochemically driven aminocatalytic asymmetric α-alkylation of aldehydes with electron-deficient benzylic bromides and phenacyl bromides **73** to yield the corresponding aldehyde products **74** in high yields and high enantioselectivities (Scheme 39) [109]. Unlike previously reported photocatalytic approaches, in this realization, the catalysts do not contain any photosensitive group. Instead, a chiral electron donor-acceptor colored EDA complex **I-10** is formed upon coordination of the in situ-generated enamine intermediate and the alkylating aryl-containing reagent. As shown in the scheme, this EDA complex may then suffer a photoinduced electron transfer with ulterior liberation of a halide anion, followed by enantioselective radical-radical coupling as in **TS-16** to give the corresponding iminium intermediate that would evolve as under common thermal conditions with regeneration of the amine catalyst. Various experimental evidences were provided in support of the in-cage radical combinations as the stereodetermining step. Formation of the EDA complexes was supported by spectrophotometric measurements in control experiments using either benzylic bromides or α-bromo acetophenone. Although Hayashi-Jørgensen prolinol derivatives were competent catalysts for this visible light-induced transformation, catalyst **C62** was found to provide superior enantioselectivities. While most of the reaction scope was developed with linear aldehydes **1** (R^1^ = H), the reaction with α-phenyl propionaldehyde also proceeded nicely to afford after 40 h product **75** in 93% yield and 86% *ee*.

**Scheme 40:**
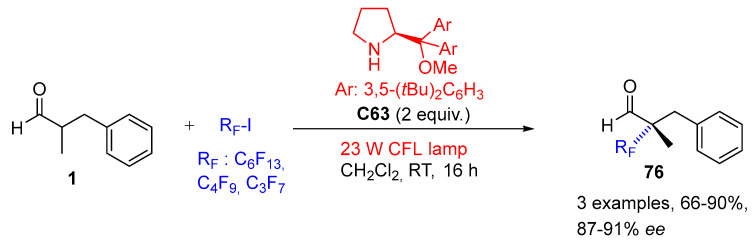
EDA complexation-induced photochemical α-perfluoroalkylation of aldehydes.

In a more recent example of EDA complexation-induced photochemical α-alkylation of aldehydes, Yajima et al. described the α-perfluoroalkylation of α-benzyl propionaldehyde **1** using diarylprolinol methyl ether **C63** as the best performing chiral amine promoters in the presence of white or blue LEDs (Scheme 40) [110]. Other types of LEDs were less efficient. The reaction in the absence of the amine did not proceed, and in the absence of light, the reactions proceeded to a very limited extent (≈6% yield). Unfortunately, this method is not truly catalytic as two equivalents of chiral amine, as the referred-to alkylating reagent was necessary.

## 7. Methods Based on Dual Enamine Activation/Brønsted Acid Catalysis

The efficiency, and sometimes the stereoselectivity as well, of aminocatalytic enantioselective α-functionalization of aldehydes may be improved when a (sub)stoichiometric amount of simple, achiral Brønsted acid additive is added. The acid additive may intervene at several elemental steps of the aminocatalytic cycle. For instance, it may ease liberation of the amine catalyst from initially formed iminium adduct, thus guaranteeing catalyst recycling. Structurally, more sophisticated, usually stronger organic Brønsted acids have been developed to trigger acid-catalyzed reactions enantioselectively [111,112,113,114,115,116,117]. In a dual activation strategy, the combined use of amine catalysts (either chiral or achiral) and Brønsted acid cocatalysts has often proved advantageous for the enantioselective α-functionalization of branched aldehydes. Here, protocols relying on this dual activation catalysis, in which the Brønsted acid cocatalyst plays a key role in the activation of the electrophilic reaction component, are presented.

**Scheme 41:**
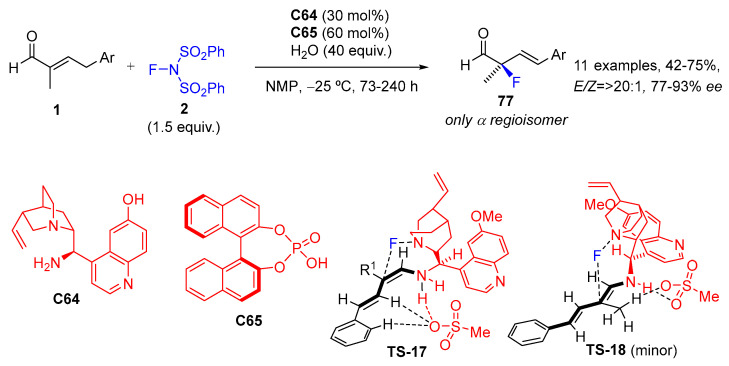
Enantioselective α-fluorination of α-substituted α,β-enals with NSFI via dienamine intermediate.

Arimitsu, Higashi et al. described regio- and enantioselective α-fluorination reactions of α-methyl α,β-enals with *N*-fluoro bis(phenylsulfonyl)imide (NSFI) via the corresponding dienamine intermediate (Scheme 41) [118,119]. Dienamines formed upon the condensation of enals with a primary amine catalyst are nucleophilic at both α- and γ-carbons. Their reaction with any suitable electrophilic reagent may thus follow two alternative pathways, the control over which is challenging. Various methodologies have been developed to promote reactions at the remote γ-position of dienamine intermediates, but successful strategies to favor a reaction at the proximal α-position are rare. Authors hypothesized that coordination of the corresponding electrophile to the catalyst quinuclidine moiety, as for **C64**, may promote a reaction at α preferentially. Catalyst screening on the aforementioned fluorination reaction with NSFI showed that primary amines bearing a quinuclidine moiety such as **C64** were superior. Adducts **77** were obtained as exclusive regioisomers in E/Z ratios of ≥20/1 and up to 93% *ee*. Participation of a Brønsted acid cocatalyst **C65** was important for achieving both good reactivity and selectivity, as was the presence of water to shorten reaction times. The stereoinduction in this reaction is highly controlled by the aminocatalyst and is slightly influenced by the configuration of the chiral Brønsted acids. For example, when methanesulfonic acid was employed as the acid cocatalyst instead of **C65,** the enantioselectivity decreased from 79% to 75% *ee* in a model reaction. DFT calculations support the key role of the quinuclidine nitrogen in switching the reactivity from the intrinsically more nucleophilic γ-carbon to the geometrically better positioned α-carbon of enamine. Calculations based on a model reaction with methanesulfonic acid predicted the favorable effect of the acid cocatalyst and water on the transition structure stabilization and the sense of stereoinduction (major **TS-17** vs. minor **TS-18**).

**Scheme 42:**
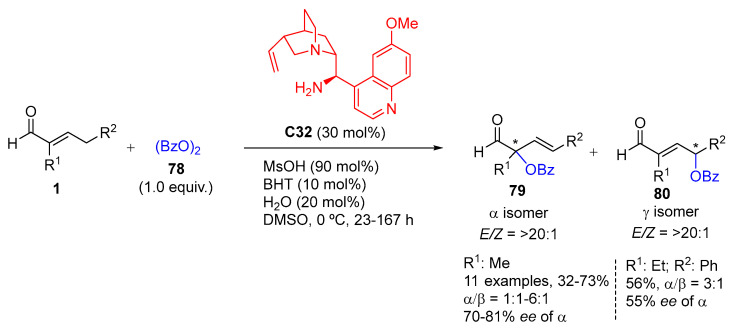
α-Benzoyloxilation of aldehydes/enals using cinchona alkaloid-derived primary amine as the catalyst and an acid cocatalyst.

In early studies, List’s laboratory reported the α-benzoyloxilation of α-branched aldehydes and of α-branched enals, respectively, with benzoyl peroxide in the presence of a cinchona alkaloid-derived primary amine as the catalyst, an acid cocatalyst, and 2,6-di-tert-butyl-4-methylphenol (BHT) as a radical scavenger [120]. In the case of enals as substrates, the α-benzoyloxilation of the putative dienamine intermediate was predominant over the γ-benzoylation process. Unfortunately, even using BINOL-derived chiral organophosphoric acid cocatalysts to enhance the stereoselectivity, the α-benzoyloxilated products were obtained with moderate enantioselectivity so far. More recently, Arimitsu reinvestigated this transformation and eventually found a synergistic effect of water and a sulfonic acid cocatalyst in DMSO as a solvent (Scheme 42) [121]. The reaction efficiency was improved, reducing the reaction time, and enantioselectivities up to 81% *ee* with excellent *E* selectivity (*E/Z* => 20/1) were obtained from α-methyl enals **1** (configuration of products not determined) using amine **C32**. As expected, the reaction with the α-ethyl analog **1** (R^1^: Et) proceeded with diminished enantioselectivity. Unfortunately, the α vs. γ regioselectivity decreased (α/γ = 1/1–6/1) as compared with that previously reported by List. At this time, authors did not provide a rationalization of the beneficial effects of the acid cocatalyst.

**Scheme 43:**
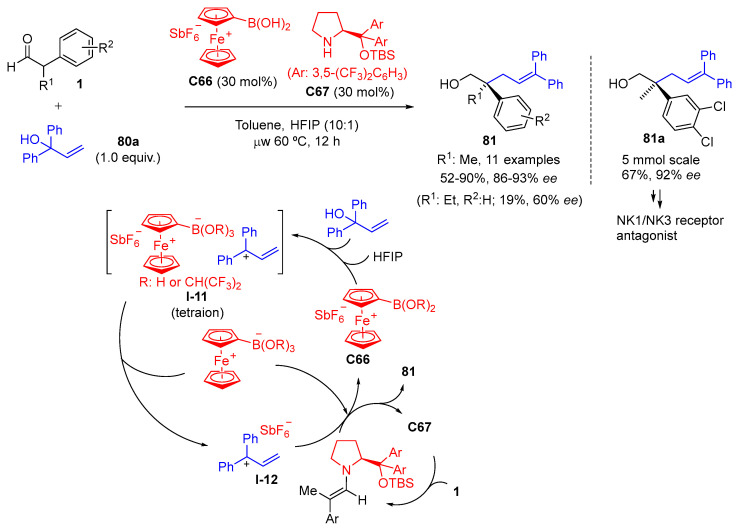
Boronic acid cocatalyzed dual catalysis strategy for allylic α-alkylation of branched aldehydes with allylic alcohols.

Hall et al. have described a boronic acid cocatalyzed dual catalysis strategy to achieve the allylic α-alkylation of branched aldehydes with allylic alcohols, a noble metal-free protocol that produces water as the only byproduct [122]. Based on the ability of boronic acids to form reversible covalent bonds with hydroxyl functionalities, various organoboronic acids in combination with *gem*-diaryl allylic alcohol **80** as the alkylating partner in order to suppress the competing boronic acid-catalyzed 1,3-rearrangement of allylic alcohols were tested. After optimization of the conditions and additives, the reaction of various α-aryl propanaldehydes with **80a** in the presence of Hayashi-Jørgensen catalysts **C67** afforded the desired linear products **81,** embedding a methyl-aryl quaternary carbon center with up to 90% yield and a 96.5:3.5 enantiomeric ratio (Scheme 43). Unlike to what is observed in many other reactions involving branched aldehydes, CF_3_-bearing diarylprolinol ethers such as **C67** performed better than the nonfluorinated analogs or the primary amine analogs. 2-Phenyl butanal (R^1^: Et) also reacted under the above conditions, although both the yield and the enantioselectivity decreased drastically. On the other hand, HFIP was critical to increase the solubility of ferrocenium boronic acid **C66,** as well as stabilize the putative carbocation intermediate **I-11**. The method was shown to be suitable for scaling up to gram quantities for the preparation of a quaternary carbon fragment of Servier’s NK1/NK3 receptor antagonist. A reaction mechanism involving two independent catalytic cycles that provide the key allylic carbocation **I-12** and the enamine intermediates which intercept each other was proposed as indicated in the scheme that features a tetraionic intermediate **I-11**.

## 8. Methods Based on Combined Use of α-Amino Acid Catalysts and a Base Cocatalyst

**Scheme 44:**
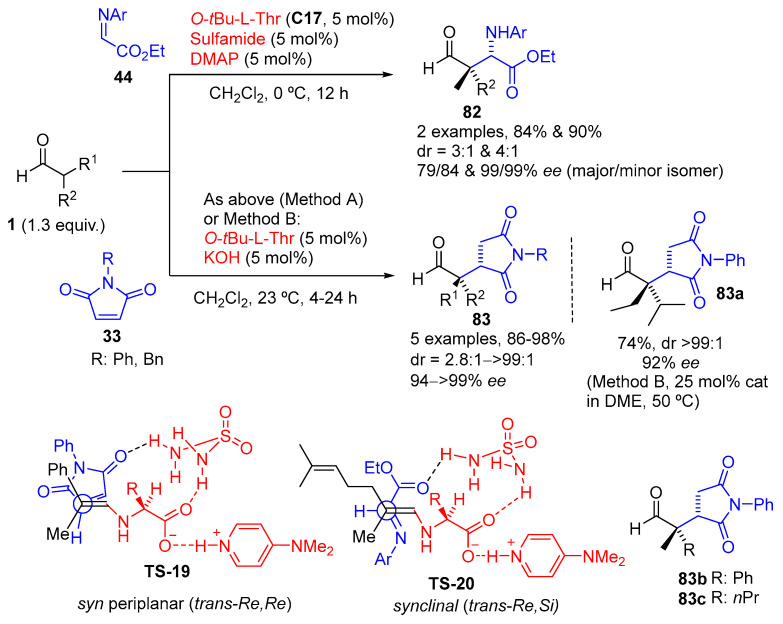
Three- and two-component self-assembled systems containing a primary α-amino acid for catalyzed Mannich/Michael addition reactions.

Enamine-mediated α-functionalization of aldehydes, in which a basic cocatalyst has beneficial effects on the course of the enantioselective transformation, have been described. In this context, Nugent et al. have described three- and two-component self-assembled systems, containing a primary α-amino acid as the key chiral element, that are able to catalyze addition reactions of branched aldehydes to *N*-aryl aldimines and *N*-alkyl/aryl succinimides, respectively (Scheme 44) [123,124]. In one variant, authors found that the system comprised of threonine *O*-*t*Bu esther **C17**, an H-bond donor (sulfamide) and a base (*N,N*-DMAP), each in 5 mol% loading, is able to efficiently catalyze the addition reaction of prostereogenic α-branched propionaldehydes to *N*-aryl aldimines **44**, affording the Mannich adducts **82** in high isolated yields, good diastereoselectivity, and high enantiocontrol. This catalytic system (Method A) may also promote the Michael addition of branched aldehydes to *N*-phenyl or *N*-benzyl succinimides **33** to give rise to adducts **83**, again in very good yields and very high diastereo- and enantioselectivity. This same Michael addition reaction may be promoted with a similar level of stereocontrol by the two-component system comprised of *O-t*Bu-L-Thr and KOH, instead. Even 2-ethyl-3-methylbutanal reacted efficiently to afford **83a** in very good yield and excellent stereoselectivity, although higher reaction temperatures and catalyst loading were required. This flexible catalyst platform, currently only based on commercially available materials, has permitted straightforward reaction fine tuning and excellent stereoselectivity for the target reaction. Supported by DFT calculations, authors have proposed a synclinal *trans-(Re,Si)* transition structure **TS-20** with a sulfamide unit bridging both reactants, the intermediate enamine and the aldimine, through H-bonding. Similarly, a *trans*-periplanar-(*Re,Re*) transition structure **TS-19** was proposed for the Michael addition to succinimides. Using this approach, sterically congested 1,2-quaternary-tertiary stereomotifs are created with unprecedented levels of stereocontrol.

Recently, the succinimide derivatives **83b**-**c** were prepared following this approach by Sadiq et al. and their inhibitory activity against cholinesterases and α-glucosidase enzymes, as well as COX-1, COX-2, and 5-LOX enzymes, and was evaluated in vitro [125,126].

**Scheme 45:**
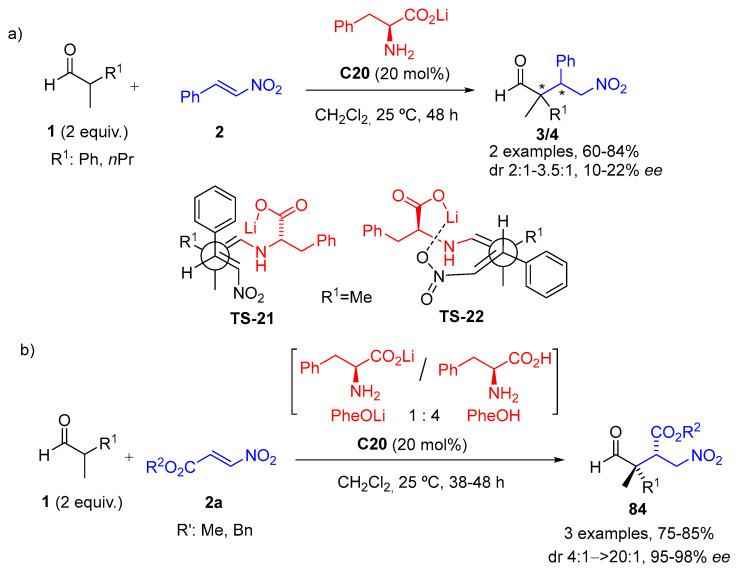
Michael additions of branched aldehydes catalyzed by (**a**) lithium salt of primary α-amino acids, (**b**) a mixture of free α-amino acid and its lithium salt.

Yoshida et al. have found that the Michael addition of branched aldehydes to *trans*-nitrostyrene may be catalyzed by the lithium salts of primary, but not secondary, α-amino acids (Scheme 45) [127]. The phenylalanine lithium salt (L-PheOLi, **C20**) was found to be the most selective. During reaction optimization, authors reported that other alkali metal salts did also promote the reaction enantioselectively, but with comparatively lower yields of product formation. In contrast, the corresponding free α-amino acids were totally inefficient. Unfortunately, with α-branched propanaldehydes **1,** the corresponding adducts **3**/**4** were obtained with modest syn/anti ratios, and only poor enantioselectivity was observed (Scheme 34a). Transition states **TS-21** and **TS-22**, based on the model previously reported by Seebach and Goliński for the addition of an enamine to a nitroolefin, were proposed as plausible.

In efforts towards expanding the reaction scope, the Yoshida group subsequently studied the use of β-nitroacrylates as acceptors, a reaction that had been previously reported by Ma group using aqueous media as solvent [128]. Reexamination of the reaction conditions eventually allowed for the discovery that enantioselectivity may be improved by adding benzoic acid as an additive. Assuming that the added acid will react immediately with the α-amino acid basic salt to produce the free α-amino acid, authors hypothesized that the actual catalytic system would consist of a mixture of free and deprotonated forms of the α-amino acid. Further refinement allowed to determine that a 4:1 mixture of free and lithium salt forms is the optimum for the reaction (Scheme 34b) [129,130]. Under these conditions, the corresponding α-quaternary aldehydes **84** were obtained in good yields and usually high diastereo- and enantioselectivity. The resulting adducts could be easily converted into 4,4-disubstituted pyrrolidine-3-carboxylic acids, including gabapentin analogues, through hydrogenolysis [131].

## 9. Enamine Mediated α-Functionalization via Reactivity Umpolung

**Scheme 46:**
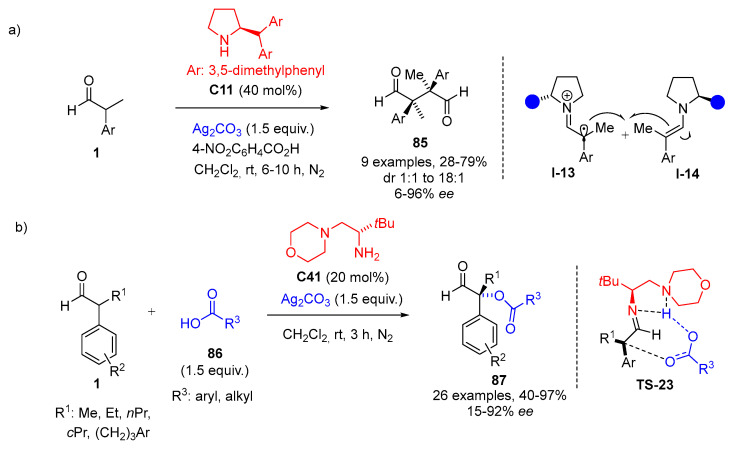
Combination of oxidant and aminocatalysts for (**a**) the homocoupling reaction of branched aldehydes, and (**b**) the aldehydes α-acyloxylation.

Enamines derived from the in situ condensation of an aldehyde and an amine catalyst are nucleophilic, so they tend to react with electrophilic species as shown in the examples above. To reverse this innate reactivity and get access to products formally derived from the coupling of an enamine and another nucleophilic reagent, various oxidative umpolung strategies have been uncovered recently [132]. In this context, in 2018, Jørgensen disclosed aminocatalytic oxidative coupling processes for α-branched aldehydes in the presence of a carboxylic acid and a source of silver(I) as the oxidant. The reactions may follow two divergent pathways, and the type of aminocatalyst employed very much determined which one is dominant (Scheme 46a,b). When using a secondary pyrrolidine-type aminocatalyst such as **C11,** complete conversion of the α-aryl propanaldehydes into the homocoupling products **85** was observed [133]. In contrast, with primary amines as catalysts, the reaction major products are the tetrasubstituted α-acyloxy aldehydes **87** [134].

In the former case, among the various oxidants and aminocatalysts tested, the combination of Ag_2_CO_3_ as the oxidant and the diarylmethyl pyrrolidine **C11** as the catalyst in dichloromethane as the solvent afforded the highest chemo- and stereoselectivity, leading to dimeric products **85** with yields from moderate to high and variable diastereo- and enantioselectivity. Aldehydes bearing electron-rich aryl substituents led to products **85** with high diastereomeric ratios and enantiomeric excesses, whereas reactions of electron-poor substrates resulted in poor stereoselectivity. In an effort to rationalize these observations, the authors investigated various alternative reaction mechanisms by measuring the ionization potentials (IP) of substrates and plausible intermediates, and also by performing competition experiments to construct Hammet plots. Based on the measured IP values and the values of the Hammet σ and ρ parameters, it was concluded that, very likely, the reactivity is governed by radical character, as indicated in the key C-C bond-forming step involving enamine and the cationic radical species **I-13** and **II-14** (Scheme 46a), whereas the diastereo- and enantioselectivity are influenced by cationic character.

The reaction using Ag_2_CO_3_ as the oxidant, but a primary amine as the catalyst instead, in the presence of a carboxylic acid led to the corresponding tetrasubstituted α-acyloxy aldehydes **87** as the major or exclusive product formed (Scheme 46b) [134]. Under optimal conditions, the reaction was quite general with respect to both coupling partners, including the aromatic and aliphatic carboxylic acids on the one side, and α-aryl α-alkyl aldehydes on the other side. Although a radical coupling pathway could not be excluded for this reaction, authors postulated the likelihood of a cationic pathway involving cationic imine species as the intermediate as in **TS-23**. The divergent behavior of primary vs. secondary aminocatalysts in the above systems was tentatively ascribed to differences in first and second ionization potentials of the corresponding enamines.

**Scheme 47:**
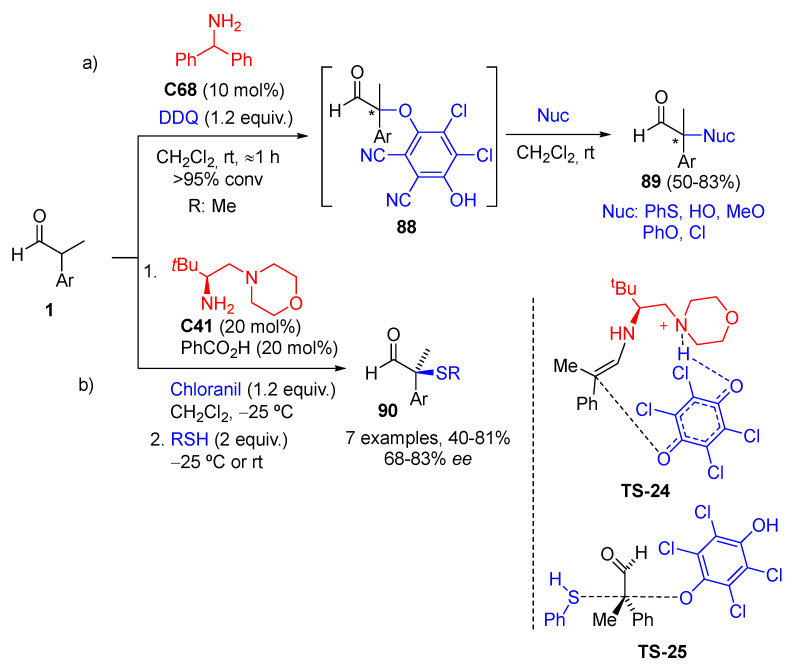
Umpolung approach for various catalytic α-oxidations (O, S, Cl) of branched aldehydes: (**a**) proof-of-concept, and (**b**) optimization of the α-aryl(alkyl)thiolation.

In a follow up development, Jørgensen and co-workers implemented a more sophisticated umpolung approach from α-branched aldehydes, wherein the initially formed α-oxy aldehydes may undergo a subsequent stereospecific S_N_2-type displacement by a suitable nucleophilic reagent. More specifically, catalytically generated enamine intermediates from α-aryl propanaldehydes react with quinones as the enabling oxidative reagent, rendering the corresponding *O*-bound α-quinol intermediate **88**. The labile *C*-*O* bond could then undergo nucleophilic displacement by a thiol reagent, allowing for a general α-thiolation of a broad selection of aldehydes in moderate to high yields (Scheme 47) [135]. Reaction optimization studies revealed that certain combinations of reactive aldehydes and quinones (e.g., 2,3-dichloro-5,6-dicyano-p-benzoquinone DDQ) proceed even in the absence of any aminocatalyst, whereas the reaction with quinones of lower reduction potential, such as chloranil (tetrachloro-p-benzoquinone) or fluoranil (tetrafluorobenzoquinone), proceeds sluggishly. Fortunately, the reaction with these latter, more accessible quinones may be boosted in the presence of benzydrylamine **C68** as the homo rising amine catalyst (Scheme 47a). Certain *O*-bound α-quinol intermediate were stable enough and their structure could be determined unambiguously. Importantly, authors demonstrated that the transformation may proceed enantioselectively when chiral primary aminocatalysts, such as the morpholine-derived amine **C41**, were employed (Scheme 47b). Control experiments indicated that the substitution step proceeds stereospecifically with complete inversion of the configuration (**TS-25**), being the overall enantioselectivity determined in the first oxidation step (**TS-24**). In addition, all experimental evidences support a polar mechanism rather than radical-mediated pathways.

**Scheme 48:**
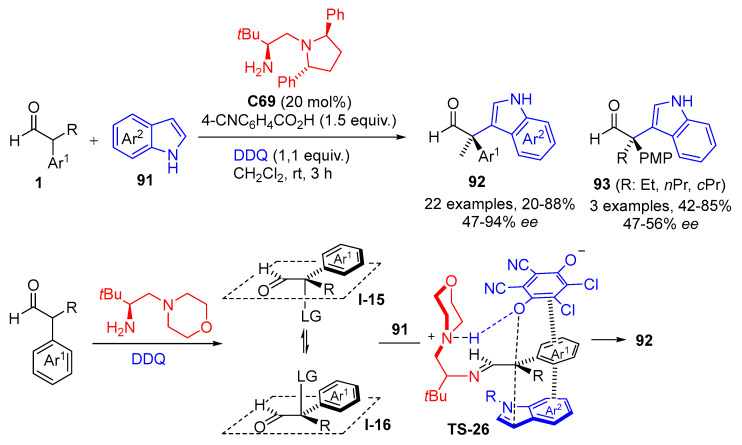
Oxidative cross-coupling of indoles with aldehydes.

In a variation of the above aminocatalytic oxidative α-functionalization of aldehydes, the Jorgensen group disclosed the first enantioselective oxidative cross-coupling of indoles with aldehydes that give access to α-quaternary aldehyde-indole conjugates in enantioselectivites of up to 94% *ee* (Scheme 48) [136]. Again, the generation of a labile α-quinol aldehyde intermediate upon oxidative coupling of the in situ-formed enamine and DDQ as the two-electron oxidant was the key, and subsequent nucleophilic displacement with indoles, which takes place through attack at the indole C-3 exclusively. Optimization of the aminocatalyst and quinone reagent for this reaction uncovered DDQ as advantageous in comparison to chloranil and fluoranil, and the primary/tertiary diamine **C69** as the most efficient catalyst in terms of enantioselectivity. If secondary amines are used as the catalyst instead, aldehydes with self-coupling adducts were obtained, probably following a one-electron oxidation pathway. A version of the method using O_2_ as the ultimate oxidant was developed, which required the participation of hydroquinone and 20 mol% of *tert*-butyl nitrite to provide the final adducts in yields and enantioselectivities comparable to those of their stoichiometric counterparts.

The above catalytic reactions with indoles are mainly limited to α-aryl propanals bearing electron-rich arenes, which provided adducts **92** in high enantioselectivities for most cases. α-Aryl aldehydes other than propanals also reacted under these conditions, albeit the corresponding adducts, i.e., **93**, were obtained in comparatively lower yields and enantioselectivity. The unfruitful search of a more general methodology prompted authors to investigate the mechanism of this transformation in some detail, which led to a rather unexpected scenario. After scrutinizing the three most plausible pathways, i.e., (i) the SOMO activation pathway followed by a second single-electron oxidation, (ii) two-electron oxidation leading to a carbocationic intermediate that would undergo S_N_1 type substitution, and finally (iii) installation of a leaving group through inner-shell two-electron oxidation by quinone and subsequent S_N_2-type displacement, combined DFT, kinetic, and empirical studies supported an atypical pathway consisting of a two-electron oxidation of the enamine intermediate, racemization of the stereolabile α-quinol intermediate, and final substitution. The process would thus imply a Curtin-Hammett regime in which the stereospecific S_N_2 also becomes stereoselective, mainly proceeding through transition state **TS-26**, effectively distinguishing between the two transient diastereomers **I-15** and **I-16**. In this process, the aminocatalyst bearing a primary amine for aldehyde condensation and a tertiary amine for hydrogen bonding is essential in each of the three elemental steps. Based on the deeper mechanistic understanding, authors identified that just 1 mol% aminocatalyst **C68** suffices for the first oxidative step with quinone. Upon full conversion to the intermediate, 20 mol% of chiral aminocatalyst **C69** was added along with the indole, allowing to maximize the aminocatalyst concentration for the S_N_2-type dynamic kinetic resolution. This adaption allowed for the generalization of the method also to electron-neutral and electron-rich aldehydes. *N*-methyl indoles were also compatible reagents, indicating minimal influence of the NH moiety [137].

**Scheme 49:**
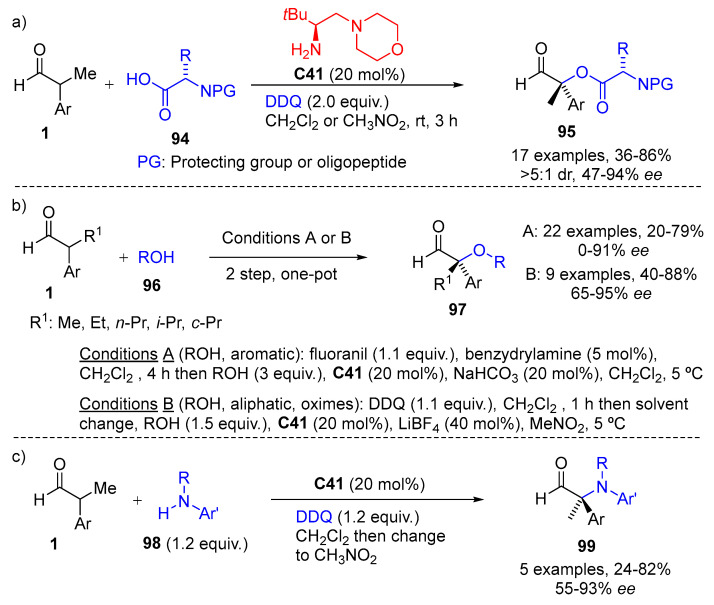
Asymmetric catalytic coupling of aldehydes with various *O*- and *N*-centered nucleophiles: (**a**) coupling with α-amino acids and oligopeptides; (**b**) coupling with alcohols; (**c**) coupling with secondary amines.

Based on this enamine-mediated oxidative umpolung coupling strategy, the Jorgensen lab has further extended this technology to coupling with various *O*- and *N*-centered nucleophilic partners, thus considerably expanding the applicability of the approach. As shown in Scheme 49, successful examples include coupling with carboxy group of α-amino acids and peptides [138], alcohols/phenols [139], and amines/hydroxylamines [140], but extension was not trivial and each nucleophile required some reaction adjustment. For instance, the bioconjugation of *N*-Boc-protected amino acids and oligopeptides with α-aryl propanaldehydes provided the corresponding carboxy-bound α-quaternary aldehydes **95** with high diastereoselectivity, the reaction with *N*-Boc proline being an exception (Scheme 49a). Interestingly, the configuration of the newly generated aldehyde α-quaternary stereocenter is fully determined by the chirality of the catalyst, which allows to obtain coupling products with either *R* or *S* Cα configuration by just changing the catalyst quirality. Based on the same reaction scheme, the aldehydes’ α-etherification could be achieved enantioselectively under modified reaction settings (Scheme 49b). In this case, initial attempts using DDQ as the oxidant resulted in suboptimal outcomes, probably because in its reduced H_2_-DDQ form, competition exists with the incoming alcoholic/phenolic partner, and in part due to aminocatalyst inactivation by the oxidant. After extensive screening, two alternative protocols, A and B, were established. In A, conducting an all at once procedure fluoranil analog led to superior results with good compromising of reaction yields and enantioselectivity without increasing too much the loading of benzydrylamine as the sacrificial aminocatalyst. In B, a two-step one-pot protocol was set so that non-catalyzed DDQ-promoted oxidation and aminocatalyzed stereoselective nucleophilic substitution steps were carried out sequentially. α-Branched aldehydes with α-substituents larger than Me (e.g., Et, *n*Pr, *i*Pr, *c*Pr) also reacted under these conditions, albeit higher benzydrylamine loading (20 mol%) was necessary and products were obtained with comparatively lower yields and enantioselectivity. The umpolung approach could also be applied to nitrogen nucleophiles, specifically aromatic amines, rendering the corresponding α-arylamino aldehydes **99** (Scheme 49c). The enantioselective version using **C41** as a catalyst led to adducts with variable yields and enantioselectivities. Still, the transformation is quite remarkable, as groups such as diarylamino can be directly attached at Cα to the aldehyde function, rendering fully substituted C-N systems. Again, changing the solvent from CH_2_Cl_2_ to CH_3_NO_2_ for the substitution step is crucial for optimal realization of a dynamic Walden-type cycle which requires rapid equilibration between the two enantiomeric quinolyl intermediates, as shown in Scheme 48.

## 10. Miscellaneous

**Scheme 50:**
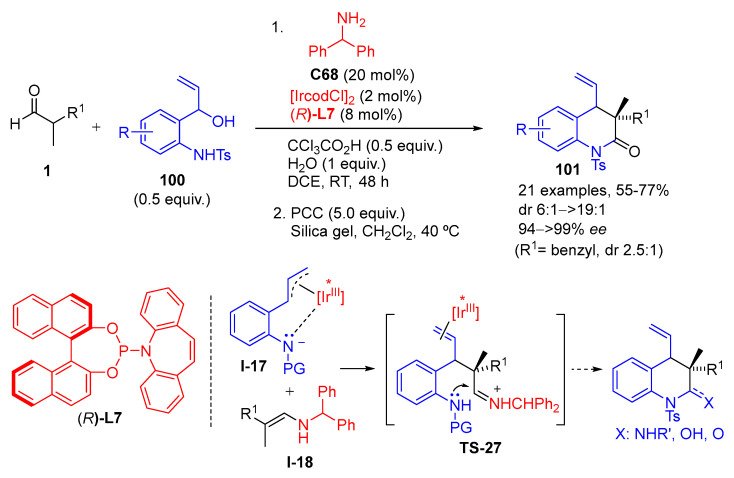
Dual catalysis conditions for the reaction between *o*-aryl-tethered vinyl aminoalcohols and aldehydes.

Lu and Xiao reported [141] the reaction between *o*-aryl-tethered vinyl aminoalcohol **100** and branched aldehydes **1** to afford 2-hydroxy dihydroquinolines, which were in situ oxidized to dihydroquinolones **101** in high yields over the two steps, high diastereoselectivity, and essentially perfect enantioselectivity under dual catalysis conditions (Scheme 50). While this method does not provide aldehydes as final products, this asymmetric α-carbofunctionalization of branched aldehydes generates synthetically versatile dihydroquinolones and allows access to a small library of derivatives in a few steps from readily available starting materials, and in a highly selective manner. The method involves two concurrent catalytic cycles, one triggered by the iridium (I)-**L7** complex, which activates the vinyl alcohol to form a cationic Ir(III) complex **I-17**, and the second one involving a primary amine (**C68**), which activates the aldehyde in the form of enamine **I-18**. The tosyl-protecting group on the starting aniline derivative was crucial, as no reaction was observed when the analog *N*-Boc or the parent *NH* substrates were used instead. The acid additive TCA had an impact on both reactivity and stereoselectivity, while water was important as a reaction accelerator. Among the tested amines, the simple achiral amine **C68** gave the best reactivity and stereoselectivity, indicating that the chiral Ir complex was responsible for the enantioinduction. The proposed course of the reaction is depicted in Scheme 50. Once the intercepted iminium species is formed, it would undergo an intramolecular amination as shown in **TS-27** to give rise to the aminal/hemiaminal product as a function of the selected workup. This isolable product then would be oxidized to the final imide product **101** in a separate, one-pot step.

**Scheme 51:**
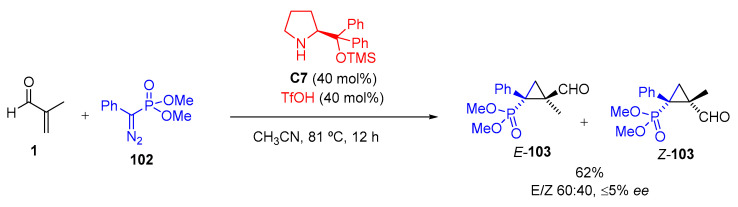
Iminium ion-mediated cyclopropanation of methacrolein with α-diazophosphonate.

Methacrolein was reported to react with α-diazobenzylphosphonate in a thermally induced cyclopropanation reaction to give rise a diastereomeric mixture of (*E*)- and (*Z*)-dimethyl (2-formyl-2-methyl-1-phenylcyclopropyl)phosphonate **103** (Scheme 51) [142]. Application of proline or proline-derived organocatalysts such as **C7** accelerated the reaction, probably by iminium ion activation mechanisms, but had a minor effect on the *E/Z* ratio of the cyclopropane product, favoring the *E*-isomer in a modest 60:40 ratio. In addition to the large amount of the catalyst required, asymmetric induction by the chiral organocatalysts was found to be negligible (0–5% *ee*) in this procedure.

## 11. Conclusions and Prospects

Aldehydes are readily available materials and the carbaldehyde functional group may undergo several transformations, including reduction to carbinol and oxidation to a carboxylic acid group, providing extraordinary synthetic versatility. Aldehydes are also present in the structure of bioactive ingredients. Therefore, technologies that allow for stereocontrolled C-H functionalization of aldehydes at the α-position have attracted great interest over time. In pursuing this goal, the ambivalent reactivity of aldehydes and the relatively high electrophilicity of the carbaldehyde group (e.g., as compared to the ketone or carboxylic esters) represents a problem. In this respect, α-branched aldehydes are among the less electrophilic because of steric protection of the carbonyl. However, this feature of α-branched aldehydes becomes a disadvantage with respect to the nucleophilic reactivity of the transient enamine and enolate intermediates generated during the reaction: Firstly, the trisubstituted enamines and enolates resulting upon treatment with an aminocatalyst or a base, respectively, would exhibit attenuated reactivity; secondly, mixtures of *E*- and *Z*-configured enamines/enolates may be formed, complicating reaction stereocontrol; and third, effective face-discrimination on a trisubstituted prostereogenic sp^2^ carbon by the incoming electrophilic reagent is not trivial. Despite these issues, in the last >15 years, several catalytic approaches for the α-functionalization of prostereogenic α-branched aldehydes that proceed in useful yields and diastereo- and enantioselectivity have been uncovered. Developments include both organocatalytic and metal-catalyzed approaches, as well as dual catalysis strategies for forging new carbon–carbon and carbon–heteroatom (C-O, N, S, F, Cl, Br, …) bond formation at Cα of the starting aldehyde. Enamine-mediated reaction development has contributed to this progress significantly. Since enamine formation is quite sensitive to steric constraints, primary amines are the preferred aminocatalysts for the α-functionalization of α-branched aldehydes, and the use of the most popular proline and other secondary amines is marginal in this context. In the majority of these aminocatalytic approaches, catalyst screening identified amines bearing an acidic sidearm (bifunctional catalysts) as optimal and the addition of a protic acid adyuvant, typically simple carboxylic acids in substoichiometric amounts, have proved beneficial in terms of both reaction conversion and stereoselectivity. A few examples of combined use of a bifunctional aminocatalyst and external base are also reported in which the actual catalyst is proposed to be the corresponding salt. The aminocatalytic, highly enantioselective α-functionalization of α-branched aldehydes has been carried out with a variety of electrophilic reagents, including oxidation, fluorination, and hydrazination reagents. In contrast, current methods for the α-chlorination and α-alkylthiolation of these aldehyde substrates proceed with lower selectivity so far. In addition, carbon–carbon bond-forming transformations can be carried out successfully, including α-alkylation/benzylation as well as additions to a range of Michael acceptors or reactive aldehydes and imines. Complementing enamine activation, catalytic methods for the α-functionalization of α-branched aldehydes that proceed through the intermediate enolate species are also reported. In this context, organocatalytic approaches relying on bifunctional Brønsted base catalysts or metal-catalyzed methods involving Rh(I)-, Zn(II)- and Pd(II)-enolates have been developed. The concept of dual catalysis or substrate activation has been successfully applied to the asymmetric α-functionalization of α-branched aldehydes, allowing to broaden the chemical and stereochemical space within reach. Particularly, the combination of amine catalysis with transition metal catalysis (metals including Pd, Ir, Ni, Sc, Au, and Cu) has enabled to use previously unsuitable reaction partners, such as allylic esters, amines and alcohols, allenamides, alkynes, or olefins. Dual activation approaches that combine enamine catalysis with photoactivation and synergistic enamine/Brønsted acid catalysis both have contributed to developing new methods, as well. Furthermore, α-functionalization of α-branched aldehydes involving a reactivity reversal that proceeds through the formation of stereolabile α-oxy (specifically α-aryloxy) species as transient intermediates amenable to undergo nucleophilic displacement have recently been achieved, opening an entry to optically active aldehyde-nucleophile adducts. In spite of the great advances on the area, most of the reported examples involve α-branched propionaldehydes as starting substrates. Protocols for the highly enantioselective α-functionalization of larger α-branched homologues are scarce, in part due to reduced reactivity, and also because of increasingly challenging enantiotopic face discrimination.

Finally, we would like to add a note of caution regarding the veracity of the reported results in the area of enantioselective catalysis, in general, and organo-catalysis in particular. Thus, as the readers have noticed, in the overwhelming number of examples discussed in this review, the reported chemical yields and, especially, enantioselectivity are oddly variable and even inconsistent with the logic of reaction mechanisms. In this regard, we would like to stress the necessity to include the Self-Disproportionation of Enantiomer (SDE) [143] studies as a part of the experimental procedure to increase the accuracy of the reported enantioselectivity. It has been profusely demonstrated that practically all types of chiral compounds, in particular derivatives of chiral amines [144,145], alcohols [146], α- [147], β-amino acids [148], and fluorine [149], are considered as SDE-phoric groups, usually being prone to exceptionally high magnitudes of the SDE phenomenon. Besides recrystallization, the SDE routinely takes place under the conditions of achiral chromatography [150] and sublimation [151,152], leading inadvertently to the variability of the recorded stereochemical data [153,154]. It is important to point out that since the last year, the Molecules requires mandatory SDE study for all submitted papers dealing with enantioselective catalysis, bio-catalysis, or isolation of chiral compounds [155,156]. One may assume that other academic journals might soon follow the Molecules’ initiative [157].

## Data Availability

No new data were created or analyzed in this study. Data sharing is not applicable to this article.
